# Tumor suppressor role of the complement inhibitor CSMD1 and its role in TNF-induced neuroinflammation in gliomas

**DOI:** 10.1186/s13046-024-03019-6

**Published:** 2024-04-01

**Authors:** Emre Can Tuysuz, Eleni Mourati, Rebecca Rosberg, Aleksandra Moskal, Chrysostomi Gialeli, Elinn Johansson, Valeria Governa, Mattias Belting, Alexander Pietras, Anna M. Blom

**Affiliations:** 1 Department of Translational Medicine, Division of Medical Protein Chemistry, Lund University, Malmö, Sweden; 2 Department of Laboratory Medicine, Division of Translational Cancer Research, Lund University, Lund, Sweden; 3 Department of Clinical Sciences, Cardiovascular Research Translational Studies, Lund University, Malmö, Sweden; 4 Department of Clinical Sciences, Division of Oncology and Pathology, Lund University, Lund, Sweden

**Keywords:** CSMD1, Glioma, TNF, Inflammation

## Abstract

**Background:**

The complement inhibitor CSMD1 acts as a tumor suppressor in various types of solid cancers. Despite its high level of expression in the brain, its function in gliomas, malignant brain tumors originating from glial cells, has not been investigated.

**Methods:**

Three cohorts of glioma patients comprising 1500 patients were analyzed in our study along with their clinical data. H4, U-118 and U-87 cell lines were used to investigate the tumor suppressor function of CSMD1 in gliomas. PDGFB*-*induced brain tumor model was utilized for the validation of in vitro data.

**Results:**

The downregulation of CSMD1 expression correlated with reduced overall and disease-free survival, elevated tumor grade, wild-type IDH genotype, and intact 1p/19q status. Moreover, enhanced activity was noted in the neuroinflammation pathway. Importantly, ectopic expression of CSMD1 in glioma cell lines led to decreased aggressiveness in vitro. Mechanically, CSMD1 obstructed the TNF-induced NF-kB and STAT3 signaling pathways, effectively suppressing the secretion of IL-6 and IL-8. There was also reduced survival in PDGFB-induced brain tumors in mice when *Csmd1* was downregulated.

**Conclusions:**

Our study has identified CSMD1 as a tumor suppressor in gliomas and elucidated its role in TNF-induced neuroinflammation, contributing to a deeper understanding of glioma pathogenesis.

**Supplementary Information:**

The online version contains supplementary material available at 10.1186/s13046-024-03019-6.

## Introduction

Gliomas, which originate from glial cells such as astrocytes, oligodendrocytes and ependymomas, account for 30% of all brain tumors and 80% of malignant brain tumors [1]. The 5-year survival rate is less than 35%, with a higher incidence in males than in females [2, 3]. All grades of diffuse gliomas extensively infiltrate the central nervous system parenchyma via white matter tracts and preexisting blood vessels [4]. Gliomas were initially graded based on histology. However, as of 2016, phenotypic and genomic characteristics of gliomas, such as isocitrate dehydrogenase 1/2 (IDH1/2) mutation status and co-deletion of 1p/19q chromosomal regions, have been incorporated into classification, grading, prognosis, and determination of response to therapy [5]. In addition, WHO has incorporated additional markers, including ATRX, CDKN2A/B, TERT, EGFR, H3.3 G34V/R, H3K27M, amplification of chromosome 7, loss of chromosome 10, and the presence or absence of necrosis in 2021 [6].

CSMD1 is a 390 kDa membrane-bound complement inhibitor whose typical mode of action is facilitated by its complement control protein (CCP) domains, and the gene encoding CSMD1 is located on chromosome 8 (8p23.1) [7]. CSMD1 consists of 14 N-terminal CUB domains separated by single CCP domains followed by 15 consecutive CCP domains. It is highly expressed in the brain including glia, neurons and vascular cells of the cerebral cortex as well as in the testis tissue [8, 9]. The tumor suppressor role of CSMD1 has been implicated in various cancers including breast, head and neck, lung, gastric and colorectal cancers [10–13]. Loss of CSMD1 expression is also associated with an increase in complement-mediated synaptic pruning by microglia, which activates inflammation-related pathways by controlling C3 and C7 [14, 15].

Inflammation is a natural response of organisms to infection, injury, and disease to maintain tissue homeostasis. Over time, scientific research has shown that inflammation may contribute to the growth of cancer cells and approximately 15% of cancer deaths are due to inflammation-induced mutations leading to malignant or neoplastic transformation [16, 17]. Inflammation can affect the composition of the tumor microenvironment (TME) and the characteristics of both tumor and stromal cells [18]. The activation of complement can induce an inflammation cascade that is regulated by cytokines secreted by cancer cells, stromal cells, and immune cells [19–21]. Thus, targeting the tumor microenvironment (TME), especially the inflammation-induced tumorigenesis regulated by the TME, could be a novel approach to cancer treatment.

Since CSMD1 is highly expressed in brain tissue, this study aimed to determine its potential role in glioma. A total of 1500 patients with glioma were analyzed from three different cohorts including The Cancer Genome Atlas (TCGA), Gene Expression Omnibus (GEO) and Chinese Glioma Genome Atlas (CGGA) revealing that downregulation of CSMD1 is linked to a poor prognosis and the activation of the neuroinflammation signaling pathway. Furthermore, overexpression of CSMD1 in glioma cell lines suppressed the aggressive phenotype and TNF/P65/IL-6 and IL-8/STAT3 pathways. Additionally, we observed reduced CSMD1 levels in mouse brain tumor models and glioma patients and *Csmd1* knockdown promoted brain tumor development in a PDGFB-induced glioma mouse model and orthotopic xenograft model. Our comprehensive analysis highlights the crucial function of CSDM1 as a tumor suppressor in attenuating glioma progression.

## Methods

### Patient datasets

All clinical and expression data of patients with glioma were obtained from public databases. Brain lower-grade glioma patient cohort (TCGA) was analyzed using CBioPortal (TCGA cohort, *n* = 530, www.cbioportal.org) [22, 23]. Data from patients with glioma from the Department of Neurology (Erasmus cohort, GSE16011, *n* = 284) were obtained from the GEO database (https://www.ncbi.nlm.nih.gov/geo/query/acc.cgi?acc=gse16011) [24]. Moreover, data from the patient cohort from Beijing Neurosurgical Institute (Beijing Cohort, http://www.cgga.org.cn/index.jsp, *n* = 693) were obtained from CGGA [25, 26]. Glioblastoma multiforme (GBM) patient cohort (TCGA) was used to determine the expression of CSMD1 according to the GBM subtypes (TCGA-GBM cohort, *n* = 619, www.cbioportal.org) Finally, a total of 325 patients with glioma from the CGGA database (Beijing Cohort, http://www.cgga.org.cn/index.jsp, *n* = 325) were used to assess the role of CSMD1 in response to temozolomide and/or radiotherapy. None of the patients from the datasets were excluded from the study. Sex and age were not included in the study design.

### Establishing the correlation between CSMD1 expression and clinicopathological characteristics of glioma

The TCGA cohort was separated into two groups based on the expression of CSMD1 (z-score of *CSMD1* expression relative to diploid cells) within the CBioPortal database. The group with a z-score > 0 was classified as “*CSMD1* high” group, while the group with z-score < 0 was classified as “*CSMD1* low” group. Various criteria including overall survival (OS) and disease-free survival (DFS), clinical characteristics, mutation enrichment, co-expression analysis, differentially expressed mRNAs and differentially expressed proteins were compared between the two groups utilizing the CBioPortal database. Differential gene expression analysis was conducted on the CSMD1 high and CSMD1 low groups within the TCGA cohort utilizing Ingenuity Pathway Analysis (IPA) (Qiagen). Fold change of genes ≥ 1 or ≤ -1 log_2_ fold change and false discovery rate (FDR) adjusted p-value (*q*-value) < 0.0001 were used in IPA. Affected canonical pathways, upstream regulators, networks, and biomarkers that were affected were evaluated.

In both the Erasmus cohort and the Beijing cohorts, patients were separated into two groups based on average CSMD1 expression in each cohort. Subsequently, OS analysis and an assessment of clinical characteristics were performed to validate the findings from the TCGA cohort. TCGA subtype analysis was carried out as previously described [27]. Briefly, the nearest centroid classifier was used for classification and Spearman’s correlation test was used to determine the *r*-value. *R*-values > 0.1 were included in the analysis.

### Cell culture, culture conditions and generation of stable expressing cell lines

Glioma cell lines H4, U-118 and U-87 were obtained from the American Type Culture Collection (ATCC). The purchased cell lines were immediately frozen after recultivation of the original aliquot and all the experiments were performed on cultures sourced from these secondary aliquots within a maximum of 12 passages. Cells were cultured in Dulbecco's modified eagle medium (DMEM) high glucose (HyClone) supplemented with 10% FBS (ATCC) and 1% penicillin–streptomycin (HyClone). Cells were transfected with a pJ509 plasmid containing either CSMD1 cDNA optimized for expression in human cells or mock pJ509-GFP plasmid using ViaFect transfection reagent (Promega) followed by antibiotic selection (0.5 µg/ml puromycin) as described [10]. Mycoplasma contamination was routinely checked in cells by Eurofins Genomics. CSMD1-overexpressing cells were coded as CSMD1, mock pJ509-GFP transfected cells were coded as Ctrl and untransfected cells were coded as (Wild type) WT throughout the manuscript.

TNF was purchased from Immunotools (Cat no: 11343015), resuspended in sterile 0.1% BSA in dH_2_O and used for subsequent experiments.

### In vivo mouse glioma model and patient glioma samples

DF-1 cells (ATCC) expressing replication-competent avian sarcoma-leukosis virus long-terminal repeat with splice acceptor (RCAS) encoding platelet-derived growth factor B (PDGFB) were intracranially injected into the neonatal brains of Nestin-tv-a (Ntv-a) mice with or without shRNA targeting P53 (shp53) to induce either low-grade glioma (LGG) or high-grade glioma (HGG) as described [28, 29]. To achieve *Csmd1* knockdown in vivo, we utilized RCAS-shRNA vectors that were prepared as previously described [30] and injected in the following combinations: RCAS-PDGFB + shGL2 (*n* = 5), RCAS-PDGFB + sh*Csmd1* #3 (*n* = 10) and RCAS-PDGFB + sh*Csmd1* #18 (*n* = 5). The experiment was terminated 220 days after injecting PDGFB + shGL2 (*n* = 5), RCAS-PDGFB + sh*Csmd1* #3 and RCAS-PDGFB + sh*Csmd1* #18. Mice without symptoms were euthanized and their brains were prepared for staining as specified in the Immunofluorescence section. For the orthotopic model, Ctrl (*n* = 9) and CSMD1-expressing clones (*n* = 12) of U-87 cells were implanted in the left cortex of newborn NOD SCID gamma (NSG) mice as 50,000 cells/mouse. The mice were sacrificed upon detection of brain tumor symptoms such as lethargy, poor grooming, weight loss, dehydration, macrocephaly, seizure, jumping and/or paralysis. Animal experiments were performed according to the European Union Directive of animal rights. Mice were randomly allocated into the control and experimental groups regardless of their sex.

LGG and GBM surgical tissues from patients were obtained from the Neurosurgery Department at Lund University Hospital. The inclusion criteria were age 18 ≥ or above, WHO performance status 0 to 2, and ability to give written informed consent.

### Proliferation and apoptosis assay

Glioma cells were seeded onto a black-sided 96-well plate (Corning) at a cell density of 2000 cells/well (for H4) or 4000 cells/well (for U-118 and U-87). To detect cell growth after seeding, the CyQUANT™ Direct Red Cell Proliferation Assay (Thermo Fisher Scientific) was utilized according to the manufacturer’s instructions. Cell growth was measured at 24, 48, 72 and 96 h. The data were normalized for each group at a time point of 24 h.

Cells were seeded onto a 6-well plate at a cell density of 1 × 10^5^ cells (for H4) and 2 × 10^5^ (for U-118 and U-87). Cells were incubated for 48 h and then deattached with TrypLE™ Express (Thermo Fisher Scientific). After washing with FACS binding buffer, cells were incubated with Zombie Aqua (Biolegend) and Annexin V (Immunotools) for 30 min to discriminate early and late apoptotic cells. The data were collected using CytoFlex (Beckman Coulter) and analyzed in FlowJo (BD).

### Migration, invasion and wound healing assays

Transwell membrane inserts (Corning-Falcon, 8 μM pore size, compatible with 24-well plate) for the migration assay and Matrigel-coated transwell membrane inserts for the invasion assay were used. In brief, 4 × 10^4^ cells were seeded in a total volume of 200 μl culture medium without FBS. The lower chamber was filled with 750 μl of culture medium containing 10% FBS. After a 20-h incubation period, cells were fixed using 4% paraformaldehyde (PFA), permeabilized with 100% methanol and stained with 0.5% crystal violet. Cells were then visualized using a microscope and migrated or invaded cells were quantified using ImageJ software.

When glioma cells reached confluency on a 6-well plate, a scratch was introduced with a sterile pipette tip and the percentage of FBS was reduced from 10 to 1%. After the wound was created, it was photographed immediately and 12 h later. The size of the wound area was determined using ImageJ and the percentage of wound closure was subsequently calculated. Relative wound closure was quantified using “{[(wound area at time 0 h) − (wound area at time 12 h)/(wound area at time 0 h)] × 100}” formula.

### Tumorsphere formation, colony formation and ALDH activity assay

Cells were seeded at a cell density of 1 × 10^5^ cells (for H4) and 2 × 10^5^ (for U-118 and U-87) in 2 ml of 3D Tumorsphere Medium XF (Sigma) onto ultralow attachment 6-well plate (Corning). One milliliter of fresh medium was added after three days. On day 6, the total number of tumorspheres was counted and the size of tumorspheres was measured using ImageJ software. Tumorspheres larger than 30 μm were included in the analysis.

Six-well plates were coated with culture medium containing 0.6% low melting temperature agarose (Sigma). Next, a top layer including 7500 cells/well in culture medium containing 0.3% low melting temperature agarose was added. One-hundred-fifty microliters of culture medium were added every 3 days. After 10 days (for H4 and U-87) or 20 days (for U-118), cells were incubated with 200 μl of culture medium containing 1 mg/ml nitroblue tetrazolium chloride (Sigma). Colonies were photographed using the ChemiDoc imaging system (Bio-Rad) and the number of colonies was counted by ImageJ software. A minimum of 50 clustered cells were defined as a colony.

Aldehyde dehydrogenase (ALDH) activity was quantified using ALDEFLUOR™ according to the manufacturer’s instructions. Briefly, 1 × 10^6^ cells were resuspended in 1 ml of ALDEFLUOR™ Assay Buffer and stained with 5 μl of Aldefluor reagent. Diethylaminobenzaldehyde (DEAB), an inhibitor of ALDH, was used as a negative control to determine the gating in the following flow cytometry analysis. All the gates for each group were determined according to their corresponding + DEAB control. Cells were incubated at 37 °C for 30 min, centrifuged at 300 × g for 5 min, resuspended in ice-cold ALDEFLUOR™ Assay Buffer and stored on ice until analysis using Beckman Coulter CytoFLEX. The data were analyzed using FlowJo.

### Cell lysates, western blotting and ELISA

Cells were lysed using lysis buffer 6 (R&D Systems) containing 1X protease and phosphatase inhibitor cocktail (Thermo Fisher Scientific). The protein concentration was determined with a Pierce BCA Protein Assay Kit (Thermo Fisher Scientific). Thirty micrograms of protein for each lane were separated using Mini-PROTEAN gels (Bio-Rad) and transferred to a PVDF membrane with a Trans-blot turbo transfer system (Bio-Rad). After 1 h of blocking at room temperature, the membranes were incubated with the appropriate primary antibody at 4 °C overnight. The next day, the membranes were washed three times with TBS/Tween (0.05%) for five minutes and then incubated with HRP-conjugated secondary antibodies. Antibody signals were detected with Immobilon Western Chemiluminescent HRP Substrate (Merck) and a ChemiDoc imaging system (Bio-Rad). The list of all primary and secondary antibodies used is available in Supplementary Table 1.

Glioma cells were lysed and fractionated into cytosolic, membrane/organelle, nucleus and cytoskeleton fractions according to the protocol from the ProteoExtract® Subcellular Proteome Extraction Kit (Merck Millipore).

Secreted IL-6 and IL-8 upon TNF treatment in supernatants were detected using Human IL-6 DuoSet ELISA (R&D Systems) and Human IL-8/CXCL8 DuoSet ELISA (R&D Systems).

### qPCR

RNA was extracted with RNAeasy Plus Mini kits (Qiagen) from H4 WT, U-118 WT and U-87 WT cells followed by cDNA synthesis using SuperScript III Reverse Transcriptase. Gene expression levels were quantified by TaqMan Gene Expression Master Mix (Applied Biosystems) using TaqMan Gene Expression Assays including *CSMD1* (Probe 1; Hs00899098_m1), *CSMD1* (Probe 2; Hs00899130_m1), *GAPDH* (Hs02758991_g1) and *β-Actin* (Hs99999903_m1). Relative mRNA levels were calculated using the 2^−∆Ct^ method. The average Ct value of *GAPDH* and *β-Actin* was used as an internal control.

### Immunofluorescence

Brain or tumor tissue from mice was embedded in optimal cutting temperature compound (OCT) (Thermo Fisher Scientific) and frozen in ice-cold isopentane. After cryosectioning, the cells were dried at room temperature for 30 min, fixed in ice-cold acetone at -20 °C, permeabilized in 0.3% Triton-X-100 (Thermo Fisher Scientific) in phosphate-buffered saline (PBS) and blocked with 2.5% fish gelatin in 0.3% Triton-x-100 in PBS. Sections were then incubated with appropriate primary antibodies at 4 °C overnight. The next day, the sections were washed with PBS and incubated with secondary antibodies for 1 h at room temperature in the dark followed by washing and mounting with Prolong glass medium with 4′,6-diamidino-2-phenylindole (DAPI). Images were acquired using an Olympus BX63 microscope, a DP80 camera, and cell-Sens Dimension v 1.12 software (Olympus Corporation, Tokyo, Japan). The CSMD1^+^ and P65^+^ fluorescent areas were calculated using ImageJ.

Patient samples were fixed with 4% PFA, cryopreserved in 0.5 M sucrose and embedded in OCT for cryosectioning as described [31]. Samples were then incubated with 5% goat serum followed by primary antibody incubation overnight at 4°C. Hoechst 33342 was used for nuclear staining. After mounting cryosections with Fluorescence Mounting Medium (Dako), photos were captured by a Zeiss LSM 710 confocal microscope.

All primary and secondary antibodies used are listed in Supplementary Table 1.

## Statistical analyses

Bars indicate the mean ± SD (SD was not shown in patient data) and symbols in each bar represent independent data points. Long-rank (Mantel-Cox) test, Spearman’s correlation test, non-parametric one-way ANOVA Kruskal–Wallis test or Mann–Whitney test were used for the patient data where applicable. Student’s t-test, one-way ANOVA or two-way ANOVA was used for normally distributed data. *q*-value or *p*-value < 0.05 was considered statistically significant. Long-rank (Mantel-Cox) test was used for symptom-free survival data of mice.

## Results

### Low expression of CSMD1 is associated with poor prognosis

**Fig. 1:**
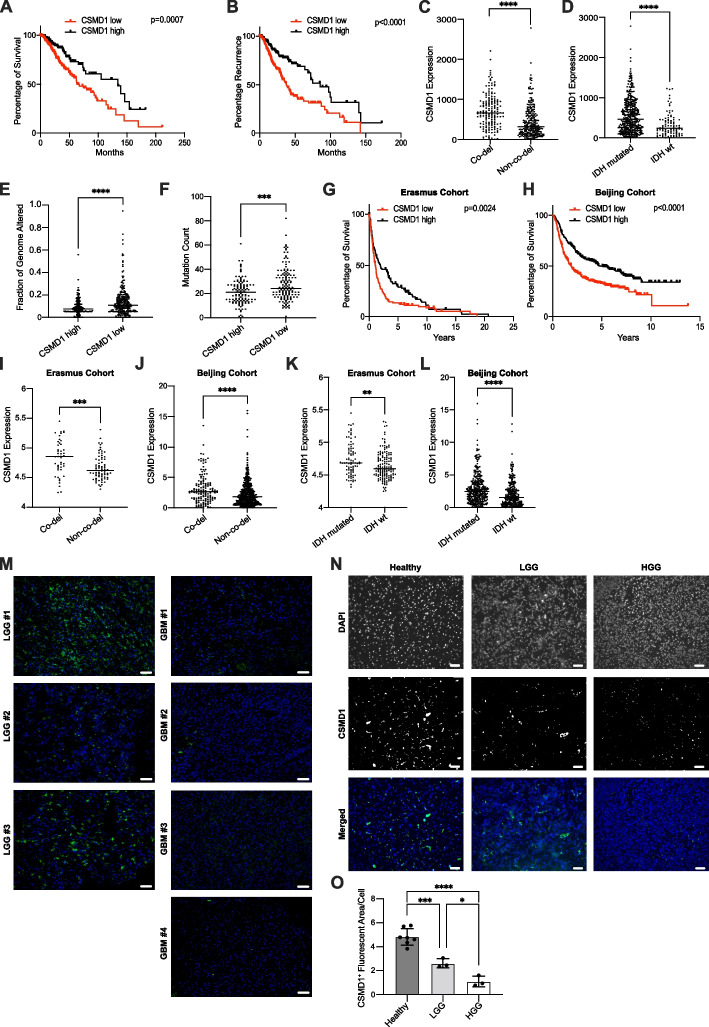
Downregulation of CSMD1 expression correlates with poor prognosis in glioma. Patients in the TCGA cohort were divided into two groups based on their expression of *CSMD1*. Downregulation of *CSMD1* expression was associated with decreased (**A**) OS time and (**B**) DFS time as well as (**C**) non-co-deleted 1p/19q and (**D**) wt-IDH phenotype in glioma samples, **E** increased fraction of genome altered and (**F**) increased mutation count in TCGA Cohort. **G**-**H** Reduced OS time in the CSMD1 low group in comparison to the CSMD1 high group and decreased expression of CSMD1 in (**I **& **J**) non-co-deleted 1p/19q and (**K**&**L**) wt-IDH were confirmed using Erasmus and Beijing cohorts. **M** Cryosections of surgical tissue sections from patients with LGG showed high expression of CSMD1 (green) while cryosections of surgical tissue sections from patients with GBM showed low or depleted expression of CSMD1. **N** The signal intensity of CSMD1 (green) was evaluated in healthy brain areas of mice as well as tumor areas from LGG and HGG samples. **O** The intensity of CSMD1 signaling per cell was found to be reduced in HGG induced by RCAS-PDGFB + RCAS-sh*p53*. Long-rank (Mantel-Cox) test was used to assess OS and DFS curves. Mann–Whitney test was used when comparing two groups (* < 0.05, ** < 0.01, *** < 0.001, **** < 0.0001) and one-way ANOVA Bonferroni’s multiple comparisons test was used when comparing 3 or more groups (* < 0.05, ** < 0.01, **** < 0.0001). wt: wild-type. Scale bar = 50 µm

Analysis of the TCGA cohort revealed that low expression of *CSMD1* in glioma is linked to a decline in both overall survival (OS) time (Fig. 1A) disease-free survival (DFS) time (Fig. 1B), non-co-deleted 1p/19q genotype (Fig. 1C) and wt-IDH phenotype (Fig. 1D). Furthermore, the fraction of genome altered (Fig. 1E) and mutation count (Fig. 1F) were lower in the CSMD1 high group than in the CSMD1 low group. The Erasmus and Beijing cohorts confirmed that reduced *CSMD1* expression correlated with decreased OS (Fig. 1G&H), absence of 1p/19q co-deletion (Fig. 1I&J) and wild-type-IDH (Fig. 1K&L) consistent with findings from the TCGA cohort. Additionally, immunohistochemical staining of sample tissues with LGG or GBM tissues obtained from the surgical resections revealed a depletion of CSMD1 expression in GBM tissues in comparison to LGG tissues (Fig. 1M). To further validate these findings, we induced glioma in mice using the RCAS/tv-a system, which mimics the clinical features of both LGG and HGG. Briefly, LGG or HGG was induced in the brains of neonatal Ntv-a mice by injecting RCAS-*PDGFB* or RCAS-*PDGFB* + RCAS-shp53, respectively. Increased cellular density, the presence of necrosis, and a shorter overall survival time confirmed that RCAS-PDGFB + RCAS-shp53 tumors were higher-grade than those induced by RCAS-PDGFB alone (Supplementary Fig. 1A-C). Further, our analysis showed that CSMD1 expression was higher in LGG than in HGG (Fig. 1N). Moreover, CSMD1 signal intensity was downregulated in both LGG and HGG samples compared to healthy brain areas (Fig. 1O).

Mutation patterns and differentially expressed genes and proteins were analyzed in two groups, CSMD1 high and CSMD1 low, using the TCGA cohort. The CIC gene mutation ratio was higher in the CSMD1 high group, whereas the PTEN and P53 mutation ratios were higher in the CSMD1 low group (Supplementary Table 2). We found differential expression of 1136 genes (756 upregulated, 380 downregulated) in the CSMD1 high group compared to the CSMD1 low group (Supplementary file 1). Moreover, TSC2, PTEN, GSK3A, GSK3B, RB1_PS807_S811 and TP53BP1 were enriched in the CSMD1 high group, while EGFR_PY1068, STAT3_PY705, MAPK1_PT202_Y204 and MAPK3_PT202_Y204 were enriched in the CSMD1 low group (Supplementary Table 3).

### CSMD1 expression correlates with decreased grade and mesenchymal subtype in patients with glioma

Our analysis indicated that *CSMD1* expression is downregulated in a grade-dependent manner in the TCGA cohort (Supplementary Fig. 2A), Erasmus cohort (Supplementary Fig. 2B) and Beijing cohort (Supplementary Fig. 2C).

CSMD1 expression was analyzed in four distinct molecular subtypes of glioma namely classical, mesenchymal, neural and proneural subtypes as defined in TCGA [27]. According to our analysis, mesenchymal subtype gliomas exhibited decreased *CSMD1* expression patterns in all three glioma cohorts (Supplementary Fig. 2D-F). In addition, receiver operating characteristic curves (ROC) were conducted for both CSMD1 expression and mesenchymal subtype in all three cohorts. In summary, the mesenchymal subtype group was compared to each of the other groups (classical, neural and proneural) separately. The ROC analysis results showed that AUC reached up to 0.8744, 0.7639 and 0.8469 in the TCGA cohort, Erasmus cohort and Beijing cohort, respectively (Supplementary Fig. 2G-O). We analyzed the expression level of CSMD1 in the subtypes of the TCGA-GBM cohort, as the mesenchymal subtype is more paramount in GBM than in LGG and we revealed that CSMD1 was downregulated in the mesenchymal GBM samples compared to other GBM subtypes (Supplementary Fig. 2P).

### CSMD1 has a tumor suppressor effect in vitro

**Fig. 2:**
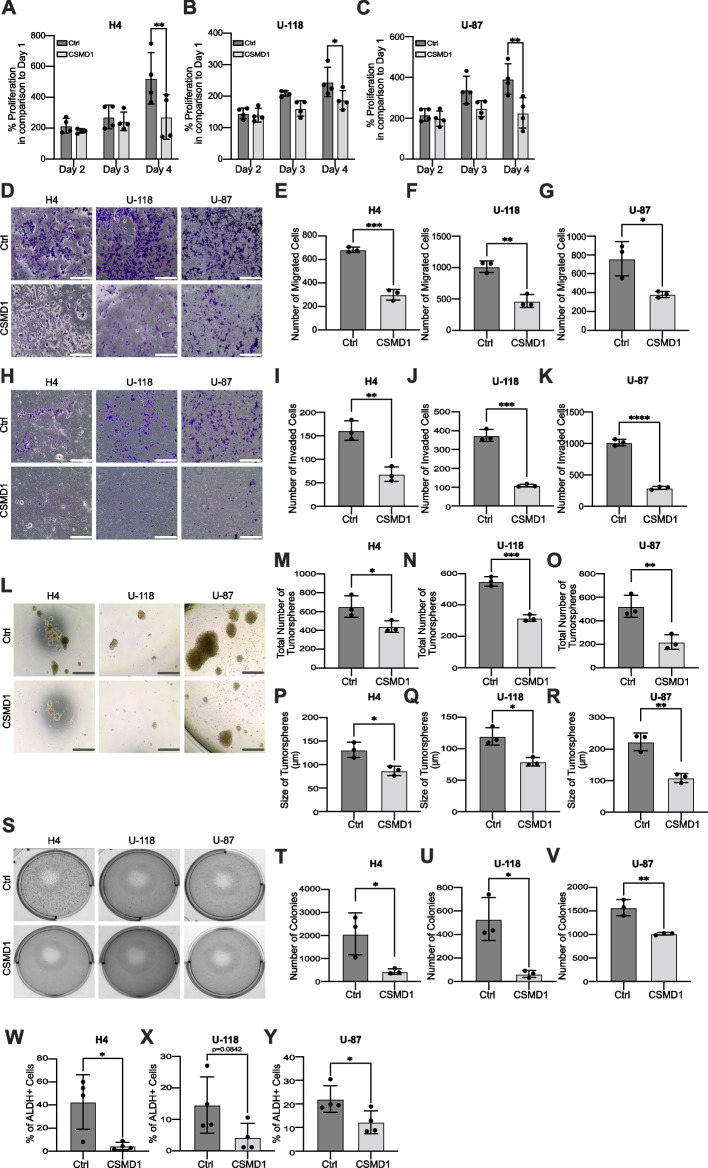
Overexpression of CSMD1 inhibited the aggressive phenotype of glioma cells. CSMD1 overexpression inhibited proliferation in (**A**) H4, (**B**) U-118 and (**C**) U-87 glioma cell lines. The data from day 1 for each group were used for normalization to accurately determine the proliferation rate. Representative images from (**D**) migration and (**H**) invasion assays. CSMD1 overexpression decreased both the (**E**–**G**) migration and (**I**-**K**) invasion abilities of glioma cells. **L** Representative images of tumorsphere formation. **M**–**O** The total number of tumorspheres was decreased with CSMD1 overexpression and (**P**-**R**) tumorspheres were smaller than Ctrl tumorspheres. **S** Representative images from the soft agar colony formation assay. **T**-**V** CSMD1 overexpression decreased the anchorage-independent growth of glioma cells in a soft agar colony formation assay. **W**-**Y** CSMD1-overexpressing cells had decreased ALDH activity in comparison to Ctrl glioma cells. Unpaired t-test was used when comparing 2 samples and two-way ANOVA Bonferroni’s multiple comparisons test was used when comparing 3 or more groups with 2 variables (* < 0.05, ** < 0.01, *** < 0.001, **** < 0.0001). Scale bar = 500 µm

Our bioinformatic analysis showed that reduced *CSMD1* expression is related to poor prognosis. To create an in vitro model to further study this phenomenon, we first detected CSMD1 expression in H4, U-118 and U-87 cells using both qPCR and western blot methods. Our analysis indicated that *CSMD1* expression was upregulated in H4 cells in comparison to U-118 and U-87 cells at the mRNA level (Supplementary Fig. 3A and B), whereas it was not detectable level at the protein level (Supplementary Fig. 3C). Ctrl and CSMD1-overexpressing clones of MDA-MB-231 cells were used as a negative and positive control in western blot experiment, respectively (Supplementary Fig. 3C). Therefore, we overexpressed CSMD1 in three glioma cell lines namely H4, U-118 and U-87 (Supplementary Fig. 3D). As depicted in Fig. 2A-C, increased CSMD1 expression effectively suppressed the proliferation of these cell lines. Furthermore, while the overexpression of CSMD1 led to a greater number of early apoptotic cells in U-118 and U-87 cells, there was no significant increase observed in H4 cells (Supplementary Fig. 3E-H). In all glioma cell lines, there were no noticeable differences in the percentage of late apoptotic cells (Supplementary Fig. 3E and I-K). Further, CSMD1 inhibited both directional migration (Fig. 2D-G) and invasion (Fig. 2H&K) in all three glioma cell lines. CSMD1-overexpressing cell lines exhibited decreased wound healing ability in all tested cell lines, while the decrease was significant only in H4 cells (Supplementary Fig. 3L-R). Tumorsphere formation and ALDH activity assays were utilized to assess cancer stem cell (CSC) transformation and CSC-like phenotype, respectively. CSMD1-overexpressing glioma cells created smaller and fewer tumorspheres (Fig. 2L-R). In addition, the expression of CSMD1 decreased the colony formation ability of glioma cells in soft agar (Fig. 2S-V). CSMD1-overexpressing cells also exhibited lower ALDH activity in comparison to their corresponding WT cells (Fig. 2W-Y and Supplementary Fig. 3S). WT cells were used instead of Ctrl cells due to GFP-positivity. While the trend was similar to that of the other two cell lines, the difference was not statistically significant in U-118 cells (*p* = 0.0842).

### CSMD1 inhibits TNF-induced NF-kB and STAT3 oncogenic signaling pathways

IPA software was utilized to determine the biological pathways moderated by *CSMD1* expression in glioma. We found that numerous pathways including neuroinflammation signaling pathway, PKCθ signaling in T lymphocytes, Th1 pathway, the complement system, role of NFAT in the regulation of immune response, iCOS9-iCOSL signaling in T helper cells, dendritic cell maturation, TREM1 signaling were inhibited in the CSMD1 high group compared to the CSMD1 low group, whereas, synaptogenesis signaling pathway, glutamate receptor signaling, cAMP-mediated signaling, calcium signaling, CREB signaling in neurons, netrin signaling and endocannabinoid neural synapse pathways were found to be more activated (Supplementary Fig. 4A). Besides, based on upstream analysis of differentially expressed genes, it was predicted that high expression of CSMD1 in glioma samples resulted in the inhibition of several cytokines that regulate inflammation including IFNG, TNF, IFNA2, IL17A, IL1A, IFNL1, IL27 and IL6 along with inhibition of NF-kB, MAP2K1/2, P38 MAPK, ERK, EGFR and MAPK14 (Supplementary Table 4). As part of our analysis, we identified potential pathways for neuroinflammation-induced cell activation. In summary, we found that pathways responsible for activating cells and regulated by TNF, CSF2, IL1A, IL17A, IL6, and IFNG were impeded in the *CSMD1* high group (Supplementary Fig. 3B-G). Finally, Supplementary Table 5 displays the significant molecules used as biomarkers for diagnosis, efficacy, prognosis, safety, response to therapy, and disease progression along with drugs that target them.

**Fig. 3:**
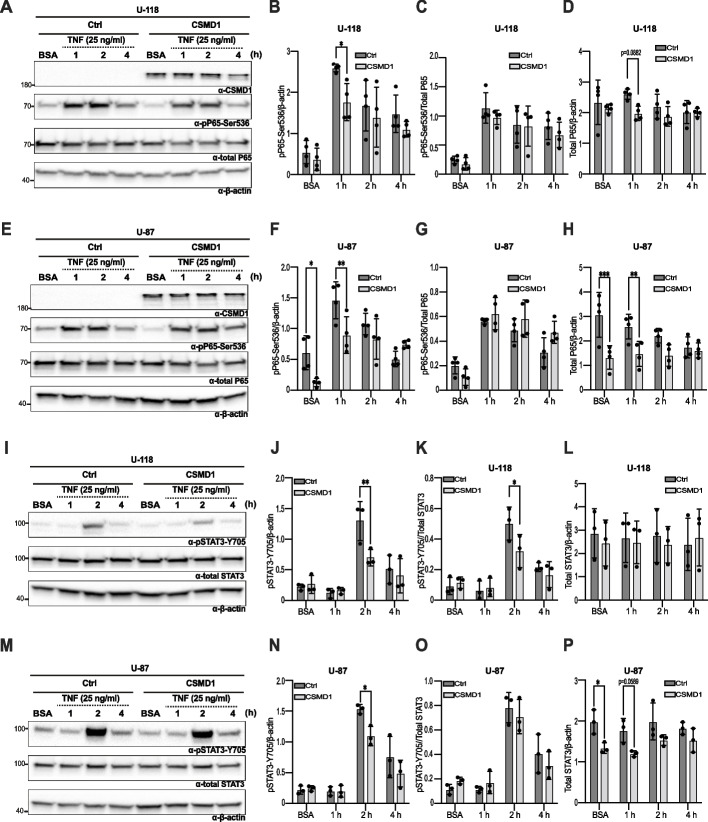
TNF-induced oncogenic signaling pathways were interrupted upon CSMD1 overexpression in glioma cells. Ctrl and CSMD1 H4, U-118 and U-87 cells were serum-starved for 2 h followed by treatment with TNF at a concentration of 25 ng/ml for 1, 2 and 4 h. A negative control was established by treating cells with BSA. **A**&**E** Representative western blots of glioma cell lines treated with BSA or TNF for the indicated time points immunodetecting pP65-Ser536, total P65 and β-actin. β-actin was used as an internal control. **B** Densitometry analysis of pP65-Ser536/β-actin, pP65-Ser536/Total P65 and Total P65/β-actin in glioma cell lines including (**B**-**D**) U-118 and (**F**–**H**) U-87. **I**&**M** Representative western blots of glioma cell lines treated with BSA or TNF for the indicated time points immunodetecting pSTAT3-Y705, total STAT3 and β-actin. β-actin was used as an internal control. Densitometry analysis of pSTAT3-Y705/β-actin, pSTAT3-Y705/Total STAT3 and Total STAT3/β-actin in glioma cell lines including (**J**-**L**) U-118 and (**N**-**P**) U-87. A two-way ANOVA Bonferroni’s multiple comparisons test was used when comparing 3 or more groups with 2 variables (* < 0.05, ** < 0.01, **** < 0.0001). h = hours

To validate findings from the bioinformatics analysis regarding neuroinflammation, we stimulated glioma cells with TNF. Firstly, we evaluated the effect of CSMD1 expression on the phosphorylation of P65 at residue Ser536, a subunit of the NF-kB complex known for its association with the response to inflammation and found that it was downregulated in CSMD1-overexpressing clones compared to Ctrl clones in all three cell lines following TNF stimulation for 1 h (Fig. 3A-H and Supplementary Fig. 5A-D). Interestingly, the overexpression of CSMD1 resulted in significantly lower ratios of pP65-Ser536/β-actin and total P65/β-actin in cells, but the pP65-Ser536/total P65 ratio remained unaltered. Furthermore, phosphorylation of STAT3 at residue Y705 was increased after TNF stimulation for 2 h (Fig. 3I-P and Supplementary Fig. 5E-H). Notably, the pSTAT3-Y705/β-actin and pSTAT3-Y705/total STAT3 (except U-87) ratios exhibited higher values in Ctrl clones of glioma cell lines than in CSMD1-overexpressing clones. The level of total STAT3/β-actin was similar among both the Ctrl and CSMD1 clones.

### TNF-induced IL-6 and IL-8 secretion is suppressed by CSMD1

**Fig. 4:**
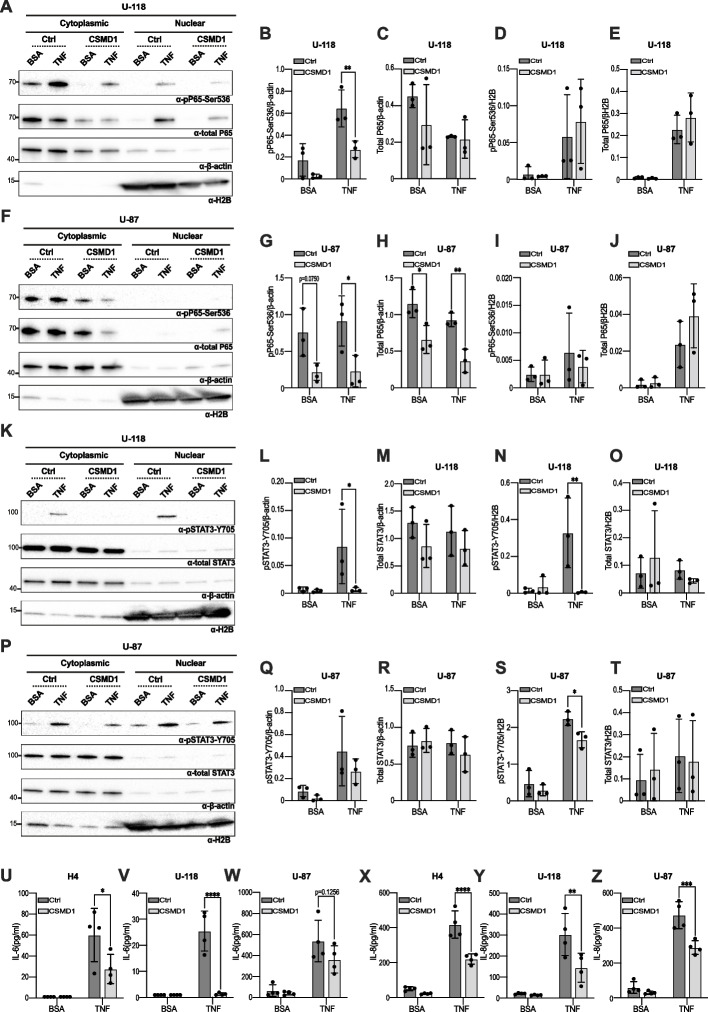
CSMD1 suppressed the activation of the NF-kB and STAT3 pathways, IL-6 and IL-8 secretion. Cells were serum-starved for 2 h followed by a treatment with TNF at a concentration of 25 ng/ml for 2 h. A negative control was established by treating cells with BSA. **A **& **F** Representative western blots of fractionated lysates of Ctrl and CSMD1-overexpressing clones of U-118 and U-87 cells immunodetecting pP65-Ser536, total P65, β-actin and H2B. β-actin was used as an internal control for the cytosolic fraction. H2B was used as an internal control for the nuclear fraction. Densitometry analysis of pP65-Ser536/β-actin, total P65/β-actin, pP65-Ser536/H2B and total P65/H2B in (**B**-**E**) U-118 and (**G**-**J**) U-87 cells after fractionation. **K** & **P** Representative western blots of fractionated lysates of Ctrl and CSMD1-overexpressing clones of U-118 and U-87 cells immunodetecting pSTAT3-Y705, total STAT3, β-actin and H2B. Densitometry analysis of p pSTAT3-Y705/β-actin, total STAT3/β-actin, pSTAT3-Y705/H2B and total STAT3/H2B in (**L**-**O**) U-118 and (**Q**-**T**) U-87 after fractionation. Cells were serum-starved for 2 h followed by a treatment with TNF at a concentration of 25 ng/ml for 2 h. A negative control was established by treating cells with BSA. The amount of secreted (**U**-**W**) IL-6 and (**X**–**Z**) IL-8 was lower in the CSMD1-overexpressing clone of the glioma cell line after BSA or TNF stimulation. A two-way ANOVA Bonferroni’s multiple comparisons test was used when comparing 3 or more groups with 2 variables (* < 0.05, ** < 0.01, **** < 0.0001)

Upon phosphorylation, the NF-kB complex is translocated from the cytoplasm to the nucleus and triggers the expression of cytokines such as IL-6 and IL-8, which act as upstream regulators of the STAT3 pathway. However, phosphorylation of the NF-κB protein does not always indicate pathway activation. To confirm pathway activation, we measured the nuclear levels of both P65 and STAT3 proteins after stimulation with BSA or TNF (25 ng/ml). A Western blot analysis performed on subfractionated U-118 and U-87 glioma cells following TNF treatment demonstrated that pP65-Ser536 was upregulated in the cytoplasmic fraction of Ctrl clones compared to CSMD1-overexpressing clones. Meanwhile, pSTAT3-Y705 was upregulated in the nuclear fraction of Ctrl clones (Fig. 4A-T). We conducted the same experiment using the H4 cell line and found that pSTAT3-Y705, but not pP65-Ser536 was upregulated in the Ctrl clone of H4 compared to CSMD1-overexpressing clones (data not shown). We subsequently quantified the levels of IL-6 and IL-8 secreted in the supernatant using ELISA after two hours of TNF treatment of glioma cells, as STAT3 was observed to be upregulated at that time point (Fig. 3I-P and Supplementary Fig. 5E-H). The quantity of both secreted IL-6 and IL-8 was higher in Ctrl clones as compared to CSMD1-overexpressing clones following TNF stimulation (Fig. 4U-Z). Since we found that phosphorylation of P65 is upregulated upon one hour of TNF treatment (Fig. 3A-H), whereas, phosphorylation of STAT3 (Fig. 3I-P), translocation of pSTAT3 to the nucleus (Fig. 4K-T) and secretion of both IL6 and IL8 (Fig. 4U-Z) were increased after two hours of TNF stimulation, we stimulated Ctrl and CSMD1-expressing clones of glioma cells with TNF at three different time points: 75 min, 90 min, and 105 min to determine the translocation kinetics of both pP65-Ser536 and total P65. Our analysis suggested that translocation of pP65-Ser536 to the nucleus was increased in the Ctrl clone of both U-118 (Supplementary Fig. 6A and B) and U-87 cells (Supplementary Fig. 6D and E) compared to their corresponding CSMD1-overexpressing clone upon TNF stimulation. Translocation of total P65 was also upregulated in the Ctrl clone of U-87 cells (Supplementary Fig. 6A and C) but not in the Ctrl clone of U-118 cells (Supplementary Fig. 6D and F) after TNF stimulation. For further validation of our in vitro data, we performed a correlation analysis between *CSMD1* expression (gene expression) versus pP65-Ser536 and pSTAT3-Y705 (at protein level) using the TCGA cohort. We revealed that CSMD1 expression was negatively correlated with both pP65-Ser536 (Supplementary Fig. 6G) and pSTAT3-Y705 (Supplementary Fig. 6H). In addition, we determined the expression level of both *IL6* and *CXCL8 (*gene expressing IL8) in the CSMD1-high and CSMD1-low groups of TCGA, Erasmus and Beijing cohorts. In line with our in vitro findings, *IL6* and *CXCL8* had reduced expression in the CSMD1-high group compared to the CSMD1-low group in all three cohorts except (Supplementary Fig. 6I-N). On the other hand, although a tendency towards reduced expression pattern was detected in the TCGA cohort for *CXCL8,* it was not statistically significant.

### CSMD1 sensitizes glioma cells to temozolomide and lomustine treatments

**Fig. 5:**
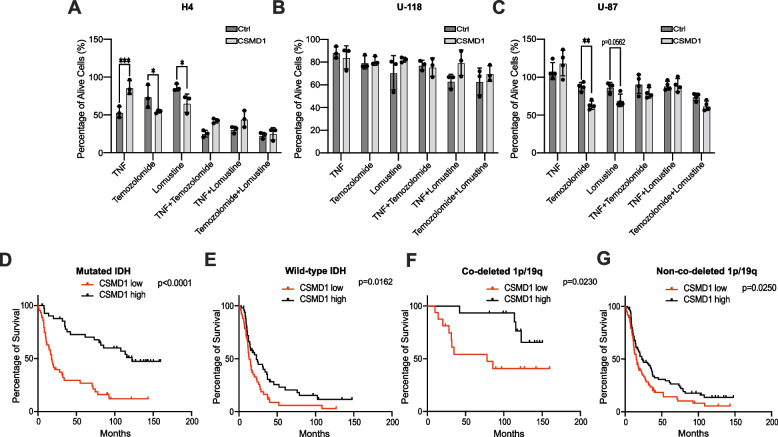
CSMD1 sensitized glioma cells to chemotherapy treatment but TNF did not increase the chemoresistance. **A** H4, **B** U-118 and **C** U-87 cells were treated with TNF (25 ng/ml), temozolomide (500 µM) and/or lomustine (50 µM) for 48 h. BSA, DMSO or BSA + DMSO treated cells were used as negative controls for normalization. High expression of CSMD1 was associated with an increased response to temozolomide treatment in (**D**) mutated IDH (*n* = 87), **E** wild-type IDH (*n* = 102), **F** co-deleted 1p/19q (*n* = 31) and **G** non-co-deleted 1p/19q (*n* = 154) glioma samples. A two-way ANOVA Bonferroni’s multiple comparisons test was used when comparing 3 or more groups with 2 variables (* < 0.05, ** < 0.01, **** < 0.0001). Long-rank (Mantel-Cox) test was used for survival curves

High-dose radiotherapy followed by temozolomide and/or lomustine administration is the primary treatment option for glioma. We hypothesized that CSMD1, previously associated with sensitivity to chemotherapy in breast cancer [32], could also function as a predictive marker for glioma chemotherapy. Therefore, we treated glioma cells with temozolomide and/or lomustine in combination with TNF and compared the response of Ctrl and CSMD1-overexpressing clones. CSMD1-overexpressing clones of H4 and U-87 glioma cells were more responsive to temozolomide treatment than their corresponding Ctrl clones, although not in U-118 glioma cells (Fig. 5A-C). Furthermore, TNF did not cause chemoresistance in any of the examined glioma cell lines. To validate our in vitro data, we subsequently used a glioma cohort from CGGA consisting of 325 patients. Upregulation of CSMD1 expression in gliomas was positively correlated with increased OS following temozolomide treatment independent of IDH mutation or 1p/19q co-deletion status (Fig. 5D-G). The impact of CSMD1 was also analyzed in the context of radiotherapy and combined radiotherapy/temozolomide treatment. The analysis showed that individuals with high CSMD1 expression had increased OS time after receiving either radiotherapy (Supplementary Fig. 7A-D) or radiotherapy with temozolomide treatment (Supplementary Fig. 7E-H) irrespective of their IDH mutation status or 1p/19q co-deletion status.

### Knockdown of CSMD1 accelerates gliomagenesis in a mouse model

**Fig. 6:**
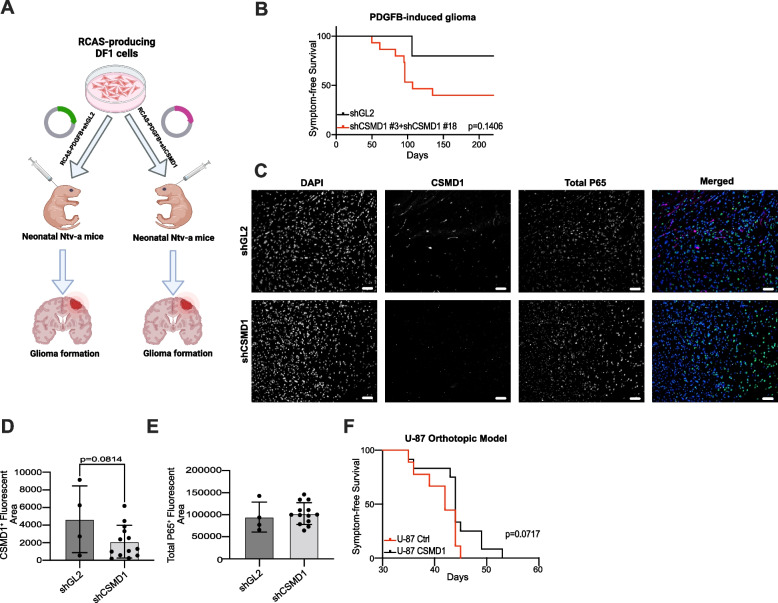
Knockdown of Csmd1 accelerated the rate of gliomagenesis in a mouse model of PDGFB-induced glioma. **A** Schematic illustration of the RCAS/tv-a system to induce glioma in mice. **B** Glial tumor formation was faster in RCAS-PDGFB + sh*Csmd1* transfected mice in comparison to RCAS-PDGFB + shGL2 mice. **C** The signal intensity of CSMD1 and total P65 immunostaining was detected in the tumor area of RCAS-PDGFB + shGL2- and RCAS-PDGFB + sh*Csmd1*-induced tumors, respectively. The Signal intensities of (**D**) CSMD1 (purple) and (**E**) total P65 (green) were detected in individual mice. **F** Ctrl and CSMD1-overexpressing clones of U-87 cells were inoculated orthotopically as 50,000 cells per mouse. Mice inoculated with CSMD1-overexpressing U-87 cells had increased survival. Long-rank (Mantel-Cox) test was used for the survival curves. An unpaired t-test was used when comparing 2 samples. Scale bar = 50 µm

To confirm the results obtained in vitro, neonatal Ntv-a mice were injected intracranially with either RCAS-PDGFB + shGL2 or RCAS-PDGFB + sh*Csmd1* (Fig. 6A). The knockdown of *Csmd1* led to a marked increase in the rate of gliomagenesis as evidenced by the formation of tumors in 9/15 animals in the shCsmd1 group in comparison with just 1/5 in the control group by the end point of the study (Fig. 6B). Moreover, glioma was detected in the brains of 3 out of 4 mice injected with RCAS-PDGFB + shGL2 and 4 out of 6 mice injected with RCAS- PDGFB + sh*Csmd1* although the mice did not exhibit any glioma symptoms. Signal intensity measurements of both CSMD1 and total P65 were also determined in the tumor region of mice injected with either RCAS- PDGFB + shGL2 or RCAS- PDGFB + sh*Csmd1*. Our analysis indicated that CSMD1 is suppressed within the tumor region of the brain injected with RCAS-PDGFB + sh*Csmd1* when compared to the tumor region of the mice injected with RCAS-PDGFB + shGL2. However, this finding did not reach statistical significance (*p* = 0.1406) (Fig. 6C and D). Further, there was no discernible difference in total P65 between RCAS-PDGFB + shGL2 and RCAS-PDGFB + sh*Csmd1*(Fig. 6C&E). Lastly, we determined whether CSMD1 expression could affect glioma formation using an orthotopic in vivo model. The orthotopic GBM model showed that mice inoculated with CSMD1-expressing clones of U-87 cells had increased survival than mice inoculated with Ctrl clones of U-87 cells with borderline significance (Fig. 6F) (*p* = 0.0717).

## Discussion

Gliomas are aggressive brain tumors that present significant challenges in treatment and prognosis despite advances in therapeutic strategies. The tumor suppressor role of CSMD1 has been demonstrated in several cancers [33] but its role in glioma has not been determined. In this study, we first revealed that elevating CSMD1 expression in glioma cell lines significantly suppressed crucial malignant traits including cancer cell proliferation, migration, invasion, and the properties linked with the cancer stem cell phenotype. Further, we discovered the effect of CSMD1 on the TNF/P65/IL-6 and IL-8/STAT3 signaling axis. The tumor suppressor function of CSMD1 was also validated using both the RCAS/tv-a brain tumor model in mice and data obtained from glioma patients. Our investigation, which includes clinical correlations, experimental models, and in vivo validations, provides a comprehensive understanding of the complex function of CSMD1 in glioma progression and illuminates its potential as a therapeutic target and prognostic marker.

The tumor mutation burden negatively correlates with OS in the gliomas [34]. Furthermore, the association of loss of *PTEN* and *TP53* with tumorigenesis is observed in most cancers. Several studies have suggested that mutations in *PTEN* or *TP53* are associated with poor prognosis, as well as shorter OS and/or higher grade in gliomas [35–37]. The mutation patterns of both *PTEN*, which is a repressor of the PI3K signaling pathway, and *TP53*, which is responsible for cell division, were higher in the CSMD1 low group in the TCGA cohort. We also showed that the knockdown of *Csmd1* in the RCAS/tv-a brain tumor model increased glioma formation in mice. Thus, reduced CSMD1 expression in gliomas could contribute to increased mutation patterns and accelerated brain tumor formation in mice.

Tumor-promoting inflammation is a hallmark of cancer [38]. Inflammation activates oncogenic signaling pathways including NF-κB, MET, STAT3, mTOR and MAPK, leading to tumor progression and an aggressive phenotype [39–42]. Various inflammatory cytokines, including TNF, CSF2, IL1A, IL17A, IL6, and IFNG, were predictively inhibited in glioma samples exhibiting high CSMD1 expression. Additionally, several other oncogenic proteins, such as RELA, STAT3, and the NFkB complex, were also inhibited. TNF, a proinflammatory cytokine secreted mainly by myeloid cells and also by cancer cells, plays a pivotal role in cancer progression and inflammation. TNF binds to its ligand TNFR1 to activate the NF-kB pathway, which is highly activated in the mesenchymal subtype of glioma [43]. CSMD1 is typically less expressed in this subtype compared to other glioma subtypes. Bioinformatic analysis revealed inhibition of the NF-kB pathway in the CSMD1-high group. The translocation of the activated NF-kB complex from the cytosol to the nucleus induces the expression of genes involved in cytokine production and the regulation of angiogenesis and apoptosis [44]. Inflammation is strongly associated with the phosphorylation of P65 at the serine-536 residue [45]. Moreover, the synergistic effect of both TNF and IL-6 exacerbates the aggressiveness of GBM by activating the NF-kB and STAT3 pathways [44]. We found that overexpression of CSMD1 in glioma cells resulted in a decrease in phosphorylation of both P65 and STAT3 as well as a decrease in secretion of both IL-6 and IL-8 when compared to the corresponding Ctrl glioma cells. The gene expression level of both *IL6* and *CXCL8* was also downregulated in the CSMD1-high group compared to the CSMD1-low group in glioma cohorts. Additionally, in the TCGA cohort, pSTAT3-Y705 was significantly upregulated in the CSMD1 low group in contrast to the CSMD1 high group and *CSMD1* expression was negatively correlated with both pP65-Ser536 and pSTAT3-Y705. Both pSTAT3-Y705 and pP65-Ser536 showed enrichment in the nuclear and/or cytoplasmic fractions of Ctrl glioma cells treated with TNF, in contrast to the CSMD1-overexpressing glioma cells. The decreased activation of the NF-kB and STAT3 pathways is likely regulated by CSMD1, although the exact mechanism requires further studies.

The complement system is part of innate immunity and its overactivation is associated with neuroinflammation and neurodegenerative diseases [46]. In glioma, complement activation is associated with poor prognosis [20]. CSMD1 functions as a complement inhibitor, while also having a direct tumor suppression effect. In vitro, overexpression of CSMD1 in glioma cell lines restrained the aggressive phenotype*.* In our in vitro system, we used heat-inactivated FBS that lacks active complement proteins. Based on this, it seems that CSMD1 holds a direct tumor suppressor function in gliomas independent of its complement-inhibitory role, as long as the glioma cell lines do not secrete substantial amounts of different complement proteins. It is noteworthy that there is an association between complement gene expression and temozolomide resistance in gliomas [47]. We observed that the overexpression of CSMD1 increased the sensitivity of the glioma cell lines, H4 and U-87, to temozolomide treatment. However, CSMD1 did not have a significant effect on U-118 cells, compared to the other glioma cell lines used in this study. Further research is needed to determine whether combining CSMD1 with human serum containing active complement proteins could improve its effectiveness in enhancing temozolomide sensitivity.

TNF did not induce chemoresistance, despite the upregulation of pP65-Ser536 being associated with temozolomide resistance in GBM [48]. This could be due to insufficient exposure time to TNF. In addition, EGFR + TNF inhibition is more effective than temozolomide treatment in glioma cells where methylguanine methyltransferase (*MGMT*) is unmethylated, whereas it is comparable in MGMT methylated cells [49]. We previously reported on the interaction between CSMD1 and EGFR in triple-negative breast cancer and found that CSMD1 overexpression led to increased cell apoptosis following treatment with gefitinib, an EGFR inhibitor [32]. Studies have indicated that *EGFR* amplification and/or mutations frequently occur in GBM but targeting EGFR has limited efficacy for GBM treatment [50, 51]. Temozolomide treatment increased the levels of proinflammatory cytokines such as TNF, IL-1β and IL-6, which are frequently linked to drug resistance, both in vitro and in vivo [52]. Consequently, a treatment plan that combines temozolomide with EGFR and TNF inhibition may represent an effective therapeutic approach for gliomas expressing CSMD1.

The knockdown of *Csmd1* promoted glioma formation in mice. Moreover, there was a clear trend for the downregulation of CSMD1 in the tumor region of mice injected with RCAS-PDGFB + sh*Csmd1* compared to RCAS-PDGFB + shGL2. Although our analysis did not reach statistical significance due to the small sample size of mice (*n* = 5) injected with the RCAS-PDGFB + shGL2 vector who developed brain tumor symptoms before the endpoint of the study, there was a clear trend toward glioma formation upon CSMD1 knockdown. Furthermore, we used an orthotopic brain tumor model for further validation of our findings from the RCAS-PDGFB brain tumor model in mice. Our analysis showed that mice inoculated with CSMD1-expressing clone of U-87 exhibited longer symptom-free survival compared to those inoculated with U-87 Ctrl cells, consistent with results from the RCAS model. These findings suggest that CSMD1 expression in glioma is a favorable phenomenon and CSMD1 functions as a tumor suppressor.

CSMD1 was downregulated in a grade-dependent manner in the studied patient cohorts, and it was nearly absent in GBM, classified as a “cold” tumor due to a lack of infiltrating T cells [53]. Moreover, a recent study suggested that TNF secretion by tumor-associated myeloid cells induces tumor-associated neutrophils with enriched proinflammatory factors that orchestrate immune-suppression and angiogenesis in glioma [54]. TNF functions by binding to two receptors, namely TNFR1, which is expressed by almost all cell types, and TNFR2, which is primarily expressed by immune cells. In addition, TNF exposure in the TME may promote an immunosuppressive phenotype through modulation of regulatory T cells and myeloid-derived suppressor cells [55]. Consequently, it could be speculated that TNF, which could be secreted by glioma cells after high-dose temozolomide treatment, is a key regulator of inflammation in the TME. Attenuated CSMD1 expression in HGG samples may modify the cytokine profile of glioma cells induced by TNF, thereby potentially aligning with the immunosuppressive phenotype in the glioma microenvironment.

## Conclusions

Taken together, CSMD1 emerges as a novel tumor suppressor in glioma that directly impacts the aggressiveness of cancer cells and regulates inflammatory pathways. These findings illuminate the potential of CSMD1 as both a therapeutic target and prognostic marker.

### Supplementary Information

**Supplementary Material 1. **

**Supplementary Material 2. **

## Data Availability

All data accessed from external sources including TCGA, CGGA and GEO have been referenced in the manuscript. Data generated or analyzed during the current study are available from the corresponding author upon reasonable request.

## Abbreviations

ALDH: Aldehyde dehydrogenase
CCP: Complement control protein
CGGA: Chinese Glioma Genome Atlas
CSC: Cancer stem cell
DAPI: 4′,6-Diamidino-2-phenylindole
DEAB: Diethylaminobenzaldehyde
DFS: Disease-free survival
DMEM: Dulbecco's modified eagle medium
FDR: False discovery rate
GBM: Glioblastoma multiforme
GEO: Gene expression omnibus
HGG: High-grade glioma
IDH1/2: Isocitrate dehydrogenase ½
IPA: Ingenuity pathway analysis
LGG: Low-grade glioma
MGMT: Methylguanine methyltransferase
NSG: NOD SCID gamma
Ntv-a: Nestin-tv-a
OCT: Optimal cutting temperature
OS: Overall survival
PBS: Phosphate-buffered saline
PFA: Paraformaldehyde
PDGFB: Platelet-derived growth factor B
RCAS: Replication-competent avian sarcoma leukosis virus long-terminal repeat with splice acceptor
ROC: Receiver operating characteristic curves
TCGA: The cancer genome atlas
TME: Tumor microenvironment
WT: Wild type
