# Supplementary material for: Tumor suppressor role of the complement inhibitor CSMD1 and its role in TNF-induced neuroinflammation in gliomas
Source: J Exp Clin Cancer Res. 2024 Apr 1;43:98. doi: 10.1186/s13046-024-03019-6 (PMC10986120; doi:10.1186/s13046-024-03019-6)
Supplement: Supplementary file 2 — Supplementary Material 2. [file 13046_2024_3019_MOESM2_ESM.pdf]

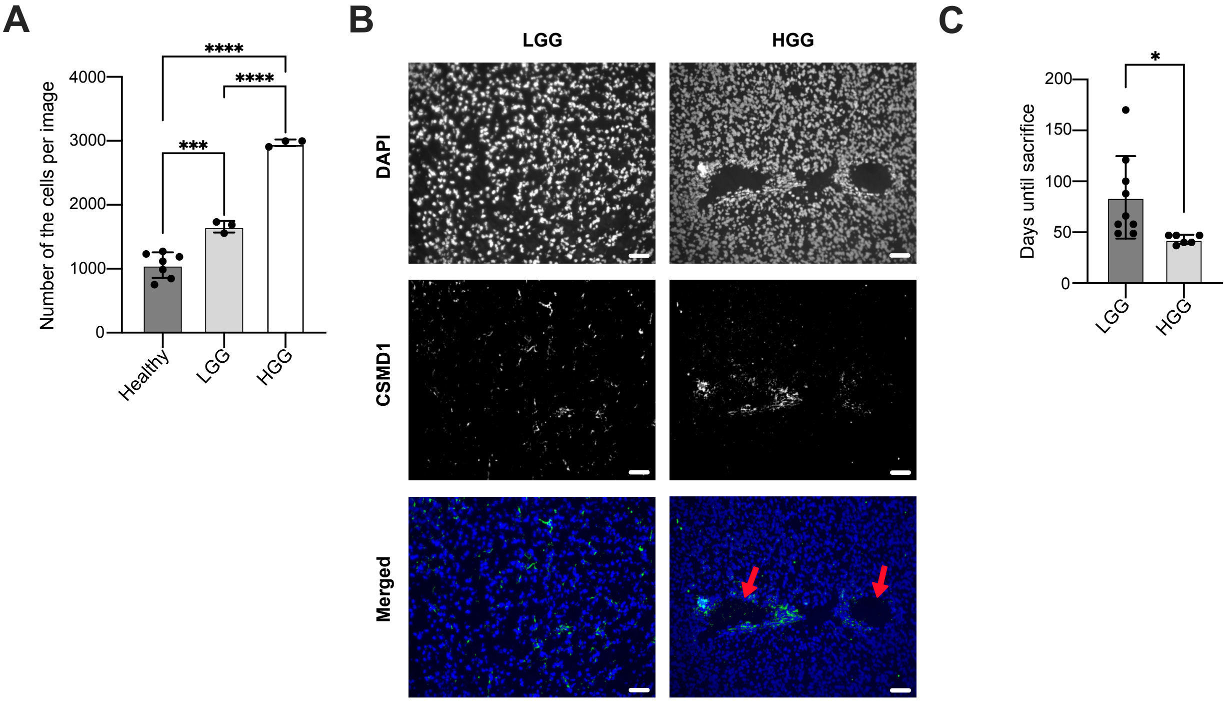

A
B
C
Number of the cells per image
4000
3000
2000
1000
0
****
****
***
Healthy
LGG
HGG
LGG
HGG
DAPI
CSMD1
Merged
Days until sacrifice
200
150
100
50
0
*
LGG
HGG

Supplementary Figure 1 Distinguishments of LGG and HGG tumors induced by RCAS-*Pdgfb* system in mice. (A) The total number of cells per image related to Figure 1N was found to be increased in HGG induced by RCAS-PDGFB+RCAS-shp53 compared to LGG. (B) Necrosis was detected only in the HGG sample (related to Figure 1N). Red arrows show the necrotic area in the HGG sample. (C) HGG induced by RCAS-PDGFB+RCAS-shp53 had a decreased average survival time than LGG induced by only RCAS-PDGFB. Data shown in C are derived from earlier experiments in which LGG and HGG were induced at different times. Unpaired t-test was used when comparing 2 samples and one-way ANOVA Bonferroni's multiple comparisons test was used when comparing 3 or more groups (* < 0.05, *** < 0.001, **** < 0.0001). Scale bar=50 μm.

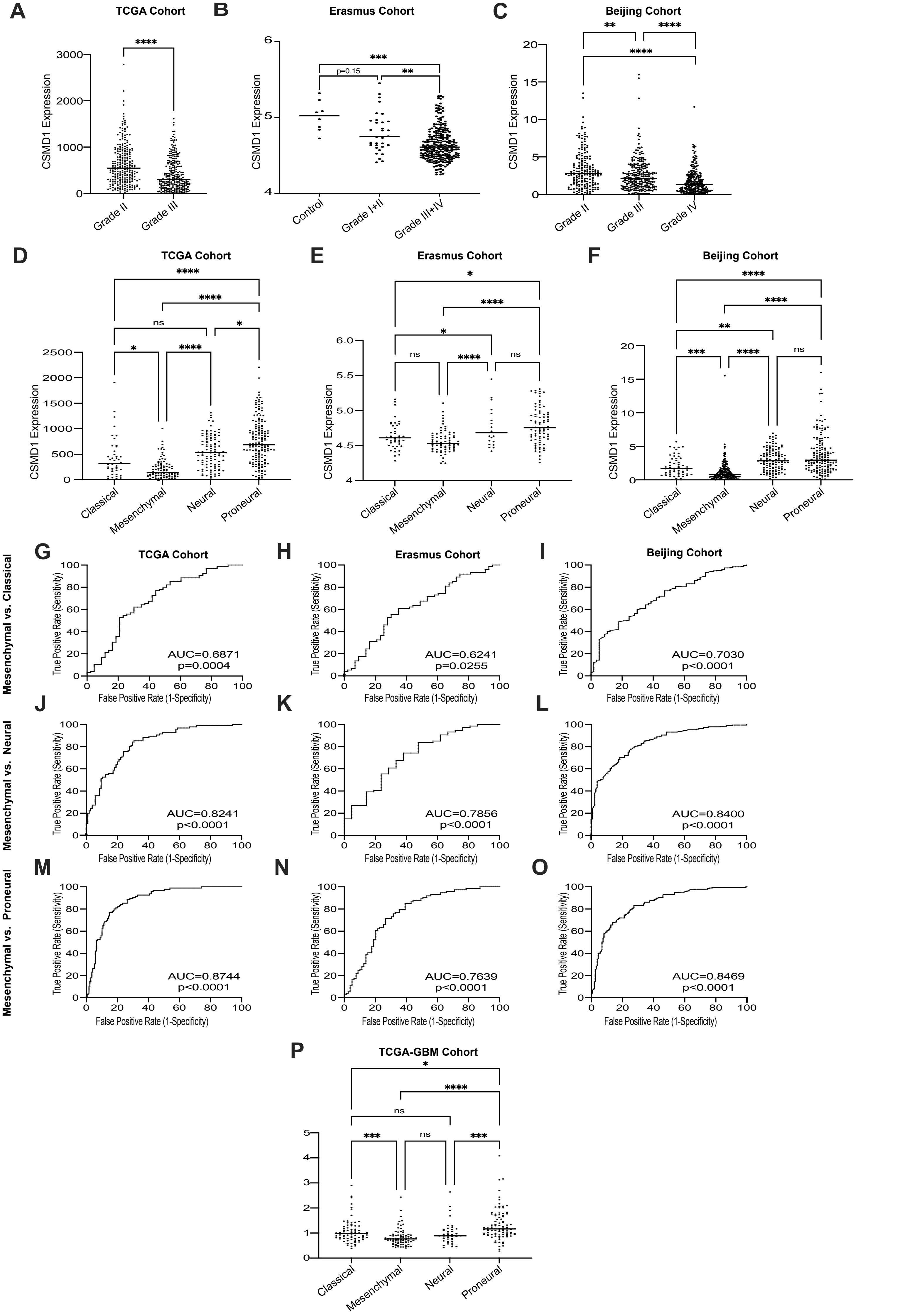

A
TCGA Cohort
CSMD1 Expression
Grade II
Grade III
B
Erasmus Cohort
p=0.15
Control
Grade I+II
Grade III+IV
C
Beijing Cohort
Grade IV
D
TCGA Cohort
E
Erasmus Cohort
F
Beijing Cohort
ns
Classical
Mesenchymal
Neural
Proneural
G
TCGA Cohort
H
Erasmus Cohort
I
Beijing Cohort
Mesenchymal vs. Classical
True Positive Rate (Sensitivity)
False Positive Rate (1-Specificity)
AUC=0.6871
p=0.0004
AUC=0.6241
p=0.0255
AUC=0.7030
p<0.0001
J
K
L
Mesenchymal vs. Neural
AUC=0.8241
p<0.0001
AUC=0.7856
p<0.0001
AUC=0.8400
p<0.0001
M
N
O
Mesenchymal vs. Proneural
AUC=0.8744
p<0.0001
AUC=0.7639
p<0.0001
AUC=0.8469
p<0.0001
P
TCGA-GBM Cohort

Supplementary Figure 2 CSMD1 expression in patients with glioma stratified by tumor grade or subtype according to gene expression signature. CSMD1 expression was downregulated in (A) TCGA, (B) Erasmus and (C) Beijing cohorts in a grade-dependent manner. CSMD1 expression among the glioma subtypes (classical, mesenchymal, neural and proneural). Attenuated expression of CSMD1 was found in mesenchymal subtypes in (D) TCGA, (E) Erasmus and (F) Beijing cohorts. (G-O) Low CSMD1 expression in gliomas can be used to predict mesenchymal subtypes in gliomas. (P) CSMD1 expression was downregulated in mesenchymal subtype GBM samples than other subtypes. Mann-Whitney test was used when comparing two groups and non-parametric one-way ANOVA Kruskal-Wallis test was used when comparing 3 or more groups (*<0.05, **<0.01, ***<0.001, ****<0.0001).

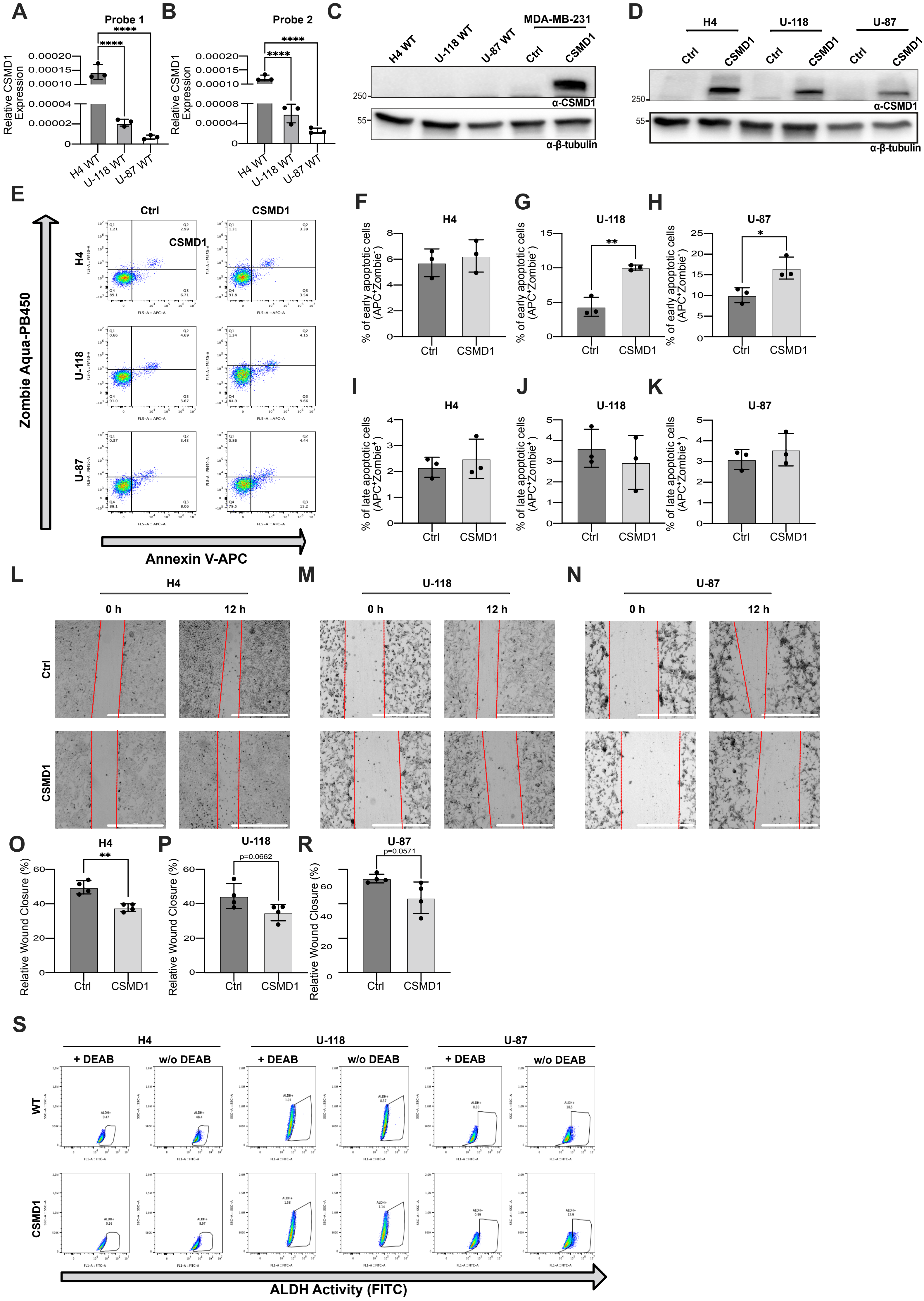
A
Probe 1
Relative CSMD1 Expression
H4 WT
U-118 WT
U-87 WT
B
Probe 2
C
MDA-MB-231
H4 WT
U-118 WT
U-87 WT
Ctrl
CSMD1
α-CSMD1
α-β-tubulin
D
H4
U-118
U-87
Ctrl
CSMD1
α-CSMD1
α-β-tubulin
E
Ctrl
CSMD1
H4
U-118
U-87
Zombie Aqua-PB450
Annexin V-APC
F
H4
G
U-118
H
U-87
% of early apoptotic cells (APC⁺Zombie⁻)
I
J
K
% of late apoptotic cells (APC⁺Zombie⁺)
L
M
N
0 h
12 h
O
P
R
p=0.0662
p=0.0571
Relative Wound Closure (%)
S
+ DEAB
w/o DEAB
WT
CSMD1
ALDH Activity (FITC)

Supplementary Figure 3 CSMD1 promoted the early apoptotic cells and inhibited the migration ability of cells. (A&B) mRNA expression level of *CSMD1.* (C) Western blot analysis of total cell lysates immunodetecting CSMD1. β-tubulin was used as a loading control. (D) Representative western blots of Ctrl- and CSMD1-overexpressing clones of glioma cell lines including H4, U-118 and U-87 immunodetecting CSMD1 and β-tubulin. β-tubulin was used as an internal control. (B) Representative dot blot of apoptosis assay 2 days after seeding the cells. (E-H) Overexpression of CSMD1 increased the percentage of early apoptotic cells in both U-118 and U-87 cells. (I-K) It did not affect the percentage of late apoptotic cells in all glioma cell lines. (L-N) Representative images of wound healing assay. (O-R) Wound healing closure in Ctrl- and CSMD1-overexpressing clones of H4, U-118 and U-87. (S) Representative dot plots of ALDH activity assay in glioma cells. Unpaired t-test was used when comparing 2 samples and one-way ANOVA Bonferroni's multiple comparisons test was used when comparing 3 or more groups (*<0.05, **<0.01). WT=WT cells, Scale bar= 500 μm.

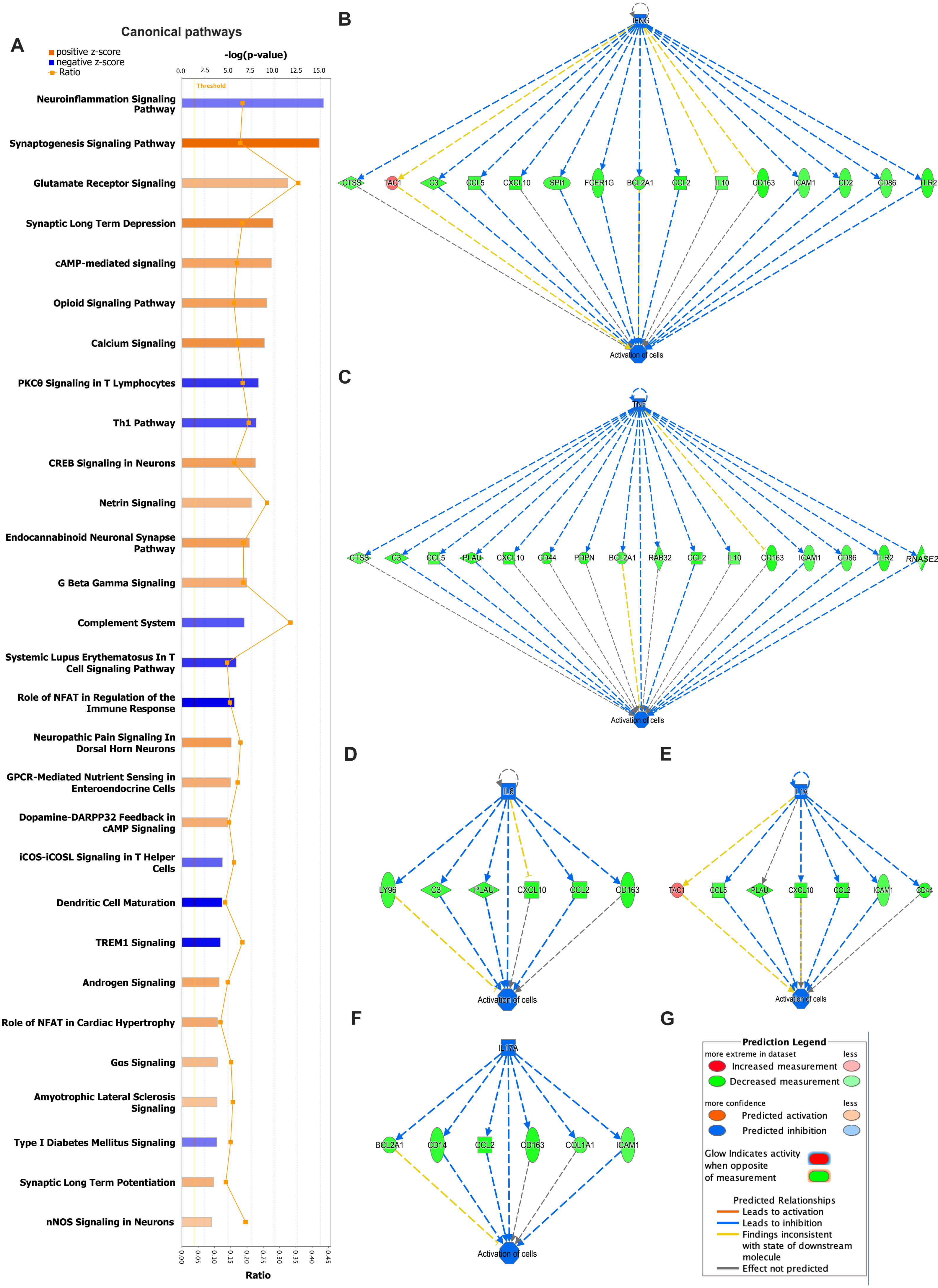

A
Canonical pathways
positive z-score
negative z-score
Ratio
-log(p-value)
0.0 2.5 5.0 7.5 10.0 12.5 15.0
Threshold
Neuroinflammation Signaling Pathway
Synaptogenesis Signaling Pathway
Glutamate Receptor Signaling
Synaptic Long Term Depression
cAMP-mediated signaling
Opioid Signaling Pathway
Calcium Signaling
PKCθ Signaling in T Lymphocytes
Th1 Pathway
CREB Signaling in Neurons
Netrin Signaling
Endocannabinoid Neuronal Synapse Pathway
G Beta Gamma Signaling
Complement System
Systemic Lupus Erythematosus In T Cell Signaling Pathway
Role of NFAT in Regulation of the Immune Response
Neuropathic Pain Signaling In Dorsal Horn Neurons
GPCR-Mediated Nutrient Sensing in Enteroendocrine Cells
Dopamine-DARPP32 Feedback in cAMP Signaling
iCOS-iCOSL Signaling in T Helper Cells
Dendritic Cell Maturation
TREM1 Signaling
Androgen Signaling
Role of NFAT in Cardiac Hypertrophy
Gαs Signaling
Amyotrophic Lateral Sclerosis Signaling
Type I Diabetes Mellitus Signaling
Synaptic Long Term Potentiation
nNOS Signaling in Neurons
0.00 0.05 0.10 0.15 0.20 0.25 0.30 0.35 0.40 0.45
Ratio
B
IFNG
CTSS TAC1 C3 CCL5 CXCL10 SPI1 FCER1G BCL2A1 CCL2 IL10 CD163 ICAM1 CD2 CD86 TLR2
Activation of cells
C
TNF
CTSS C3 CCL5 PLAU CXCL10 CD44 PDPN BCL2A1 RAB32 CCL2 IL10 CD163 ICAM1 CD86 TLR2 RNASE2
Activation of cells
D
IL6
LY96 C3 PLAU CXCL10 CCL2 CD163
Activation of cells
E
IL1A
TAC1 CCL5 PLAU CXCL10 CCL2 ICAM1 CD44
Activation of cells
F
IL17A
BCL2A1 CD14 CCL2 CD163 COL1A1 ICAM1
Activation of cells
G
Prediction Legend
more extreme in dataset
less
Increased measurement
Decreased measurement
more confidence
less
Predicted activation
Predicted inhibition
Glow Indicates activity when opposite of measurement
Predicted Relationships
Leads to activation
Leads to inhibition
Findings inconsistent with state of downstream molecule
Effect not predicted

Supplementary Figure 4 Affected canonical pathways regulated by CSMD1. (A) Inhibited (shown as blue) and activated (shown as orange) canonical pathways in CSMD1 high group compared with CSMD1 low group in TCGA cohort according to differentially expressed genes between these two groups. z-score>2 and <-2 values were used as cutoff values. Network analysis suggested that neuroinflammation signaling pathways regulated by (B) IFNG, (C) TNF, (D) IL6, (E) IL1A and (F) IL17A promoting activation of cells were inhibited by CSMD1. (G) Prediction legend for Figure 2B-F.

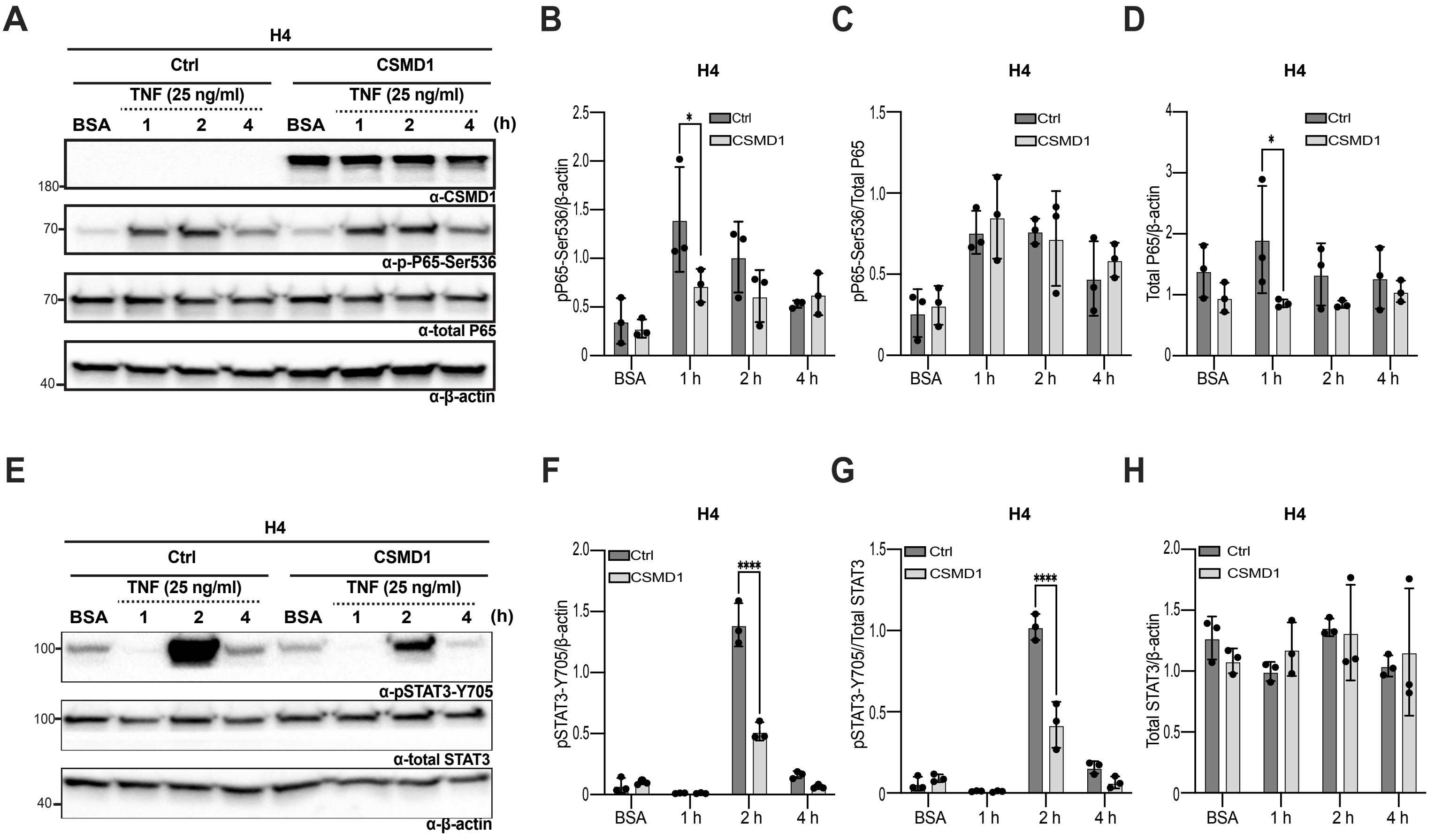
A
H4
Ctrl
CSMD1
TNF (25 ng/ml)
BSA
1
2
4
(h)
180
α-CSMD1
70
α-p-P65-Ser536
α-total P65
40
α-β-actin
B
H4
pP65-Ser536/β-actin
Ctrl
CSMD1
*
1 h
2 h
4 h
C
pP65-Ser536/Total P65
D
Total P65/β-actin
E
100
α-pSTAT3-Y705
α-total STAT3
F
pSTAT3-Y705/β-actin
****
G
pSTAT3-Y705//Total STAT3
H
Total STAT3/β-actin

Supplementary Figure 5 CSMD1 suppressed the phosphorylation of P65 and STAT3 pathway in H4 cells. Ctrl and CSMD1-overexpressing clones of H4 cells were serum starved for 2 hours and then treated with 25 ng/ml of TNF for 1, 2 and 4 hours. BSA-treated cells were used as a negative control. (A) Representative western blots of glioma cells treated with BSA either TNF for indicated time points immunodetecting p-P65-Ser536, total P65 and β-actin. β-actin was used as an internal control. (B-D) Densitometry analysis of p-P65-Ser536/β-actin, p-P65 Ser536/Total P65 and Total P65/β-actin in H4 glioma cell line. (E) Representative western blots of glioma cells treated with BSA either TNF for indicated time points immunodetecting pSTAT3-Y705, total STAT3 and β-actin. β-actin was used as an internal control. (F-H) Densitometry analysis of pSTAT3-Y705/β-actin, pSTAT3-Y705/Total STAT3 and Total STAT3 /β-actin in H4 glioma cell line. A two-way ANOVA Bonferroni's multiple comparisons test was used when comparing 3 or more groups with 2 variables (* < 0.05, ** < 0.01, **** < 0.0001)

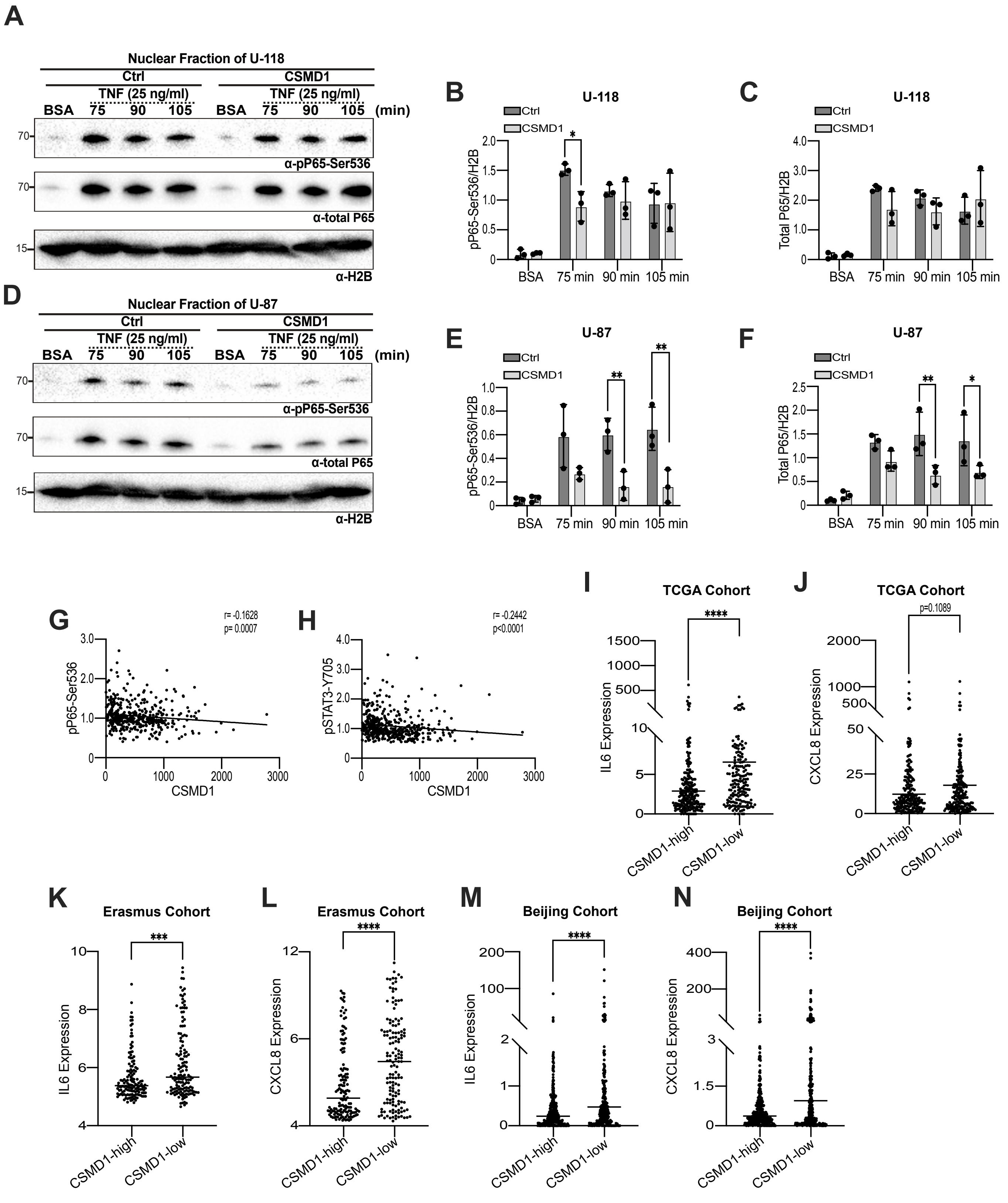
A
Nuclear Fraction of U-118
Ctrl
CSMD1
TNF (25 ng/ml)
BSA 75 90 105 BSA 75 90 105 (min)
α-pP65-Ser536
α-total P65
α-H2B
B
U-118
Ctrl
CSMD1
pP65-Ser536/H2B
BSA 75 min 90 min 105 min
C
U-118
Total P65/H2B
D
Nuclear Fraction of U-87
E
U-87
F
U-87
G
pP65-Ser536
CSMD1
r= -0.1628
p= 0.0007
H
pSTAT3-Y705
r= -0.2442
p<0.0001
I
TCGA Cohort
IL6 Expression
CSMD1-high
CSMD1-low
J
TCGA Cohort
p=0.1089
CXCL8 Expression
K
Erasmus Cohort
IL6 Expression
L
Erasmus Cohort
CXCL8 Expression
M
Beijing Cohort
IL6 Expression
N
Beijing Cohort
CXCL8 Expression

Supplementary Figure 6 CSMD1 inhibited the translocation of p-P65-Ser536 from cytoplasm to nucleus upon TNF stimulation. Ctrl and CSMD1-overexpresssing clones of both U-118 and U-87 cells were serum starved for 2 hours followed by 25 ng/ml of TNF treatment for 75, 90 and 105 minutes. BSA-treated cells were used as a negative control. (A&D) Representative western blots of nuclear fraction of Ctrl and CSMD1-overexpressing clones of U-118 and U-87 cells immunodetecting pP65-Ser536, total P65 and H2B. H2B was used as an internal control for the nuclear fraction. Densitometry analysis of pP65-Ser536/H2B and total P65/H2B in the nuclear fraction of (B&C) U-118 and (E&F) U-87 cells after TNF stimulation for indicated time points. There was a negative correlation between CSMD1 expression vs (G) pP65-Ser536 and (H) pSTAT3-Y705 in the TCGA patient cohort. Gene expression level of both (I, K & M) IL6 and (J, L &N) CXCL8 was downregulated in the TCGA, Erasmus and Beijing cohorts. Two-way ANOVA Bonferroni's multiple comparisons test was used when comparing 3 or more groups with 2 variables (* < 0.05, ** < 0.01). Spearman's correlation test was used to determine the r-value and p-value. Mann-Whitney test was used when comparing two groups (*<0.05, **<0.01, ***<0.001, ****<0.0001). min=minutes.

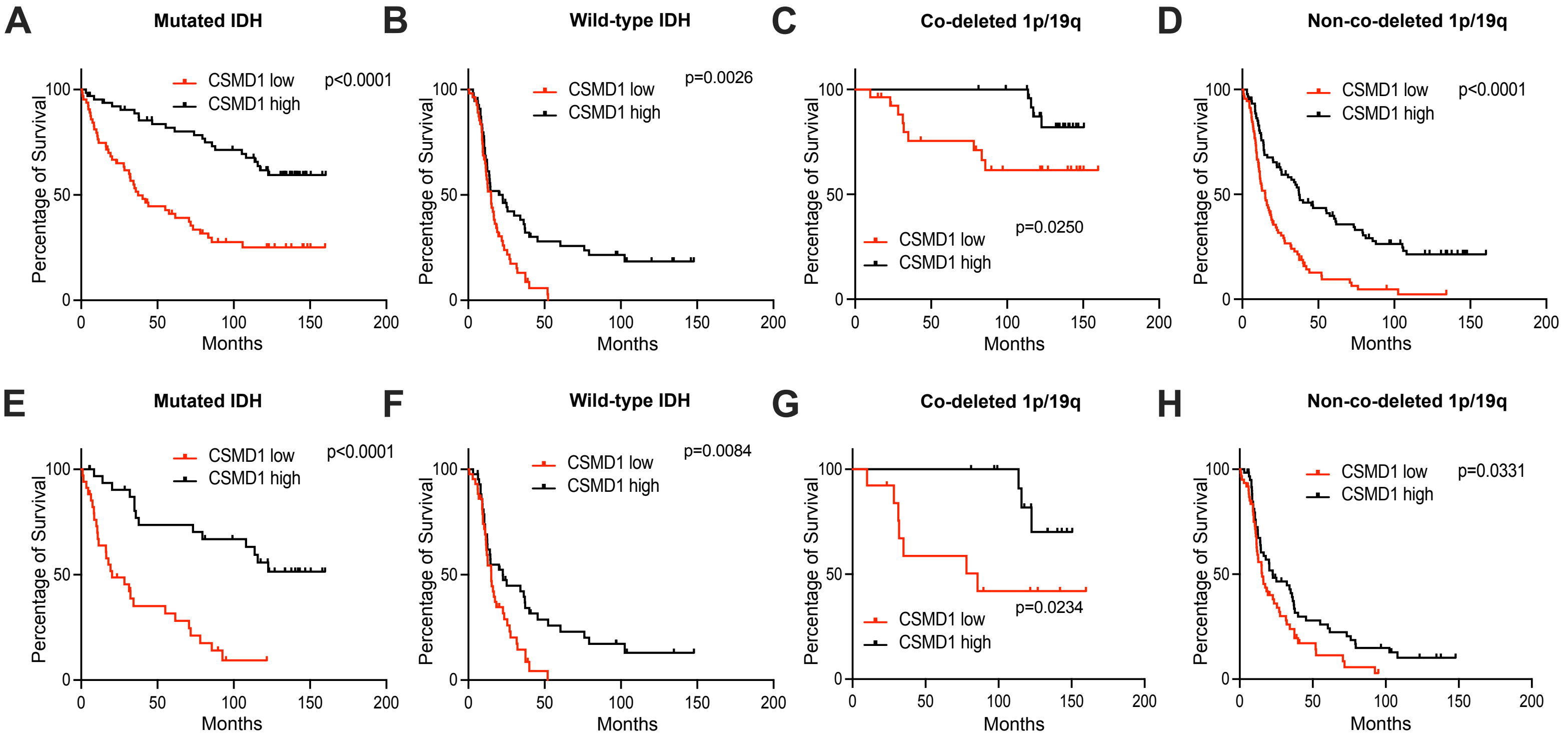
A
Mutated IDH
CSMD1 low
CSMD1 high
p<0.0001
Percentage of Survival
Months
B
Wild-type IDH
CSMD1 low
CSMD1 high
p=0.0026
Percentage of Survival
Months
C
Co-deleted 1p/19q
CSMD1 low
CSMD1 high
p=0.0250
Percentage of Survival
Months
D
Non-co-deleted 1p/19q
CSMD1 low
CSMD1 high
p<0.0001
Percentage of Survival
Months
E
Mutated IDH
CSMD1 low
CSMD1 high
p<0.0001
Percentage of Survival
Months
F
Wild-type IDH
CSMD1 low
CSMD1 high
p=0.0084
Percentage of Survival
Months
G
Co-deleted 1p/19q
CSMD1 low
CSMD1 high
p=0.0234
Percentage of Survival
Months
H
Non-co-deleted 1p/19q
CSMD1 low
CSMD1 high
p=0.0331
Percentage of Survival
Months

Supplementary Figure 7 Prognostic effect of CSMD1 expression on temozolomide treatment and/or radiotherapy. High expression of CSMD1 was associated with increased response to radiotherapy in (A) Mutated IDH (n=129), (B) wild-type IDH (n=110), (C) co-deleted 1p/19q (n=55) and (D) non-co-deleted 1p/19q (n=181) glioma samples. High expression of CSMD1 was also related to increased response to combined temozolomide with radiotherapy in (E) Mutated IDH (n=67), (F) wild-type IDH (n=86), (G) co-deleted 1p/19q (n=27) and (H) non-co-deleted 1p/19q (n=122) glioma samples. Long-rank (Mantel-Cox) test was used for survival curves.

Supplementary Table 1: List of antibodies used in the study. WB: Western blotting, IF: Immunofluorescence.

ANTIBODY	COMPANY	DILUTION	CATALOGUE NO
Anti-CSMD1	Abcam	1:5000 (WB)<br>1:250 (IF)	ab166908
Anti-β-actin	Abcam	1:5000 (WB)	ab8226
Anti-p-P65(Ser536)	CST	1:2000 (WB)	3033S
Anti-Total P65	CST	1:2000 (WB)	8242S
Anti-pSTAT3(Tyr705)	CST	1:2000 (WB)	9145S
Anti-Total STAT3	CST	1:2000 (WB)	12640S
Polyclonal Goat Anti-Mouse IgG/HRP	DAKO	1:5000 (WB)	P0447
Polyclonal Goat Anti-Rabbit Ig/HRP	DAKO	1:5000 (WB)<br>1:100000 (WB of H2B)	P0448
Polyclonal Goat Anti-rabbit IgG/HRP	Cell Sign. Tech.	1:4000 (WB)	7074
Anti-Total P65	R & D Systems	1:100 (IF)	AF5078
Anti-H2B	Abcam	1:100000 (WB)	ab1790
Goat anti-rabbit Alexa 488	Thermo Fisher	1:500 (IF)	A32731
Donkey anti-sheep Alexa 488	Thermo Fisher	1:500 (IF)	A11015
Donkey anti-rabbit Alexa 647	Abcam	1:500(IF)	ab150075

Supplementary Table 2 Mutation pattern enriched in CSMD1 high and low group. Mutation pattern of TCGA cohort was determined according to CSMD1 expression. q<0.05 was considered as statistically significant.

Gene	Cytoband	(A) CSMD1 high	(B) CSMD1 low	Log Ratio	q-Value	Enriched in
**CIC**	19q13.2	40 (33.06%)	16 (9.70%)	1.77	4.398e-3	CSMD1 high
**TP53**	17p13.1	44 (36.36%)	102 (61.82%)	-0.77	0.0257	CSMD1 low
**PTEN**	10q23.31	0 (0.00%)	13 (7.88%)	<-10	0.0257	CSMD1 low

Supplementary Table 3 Differentially expressed proteins in CSMD1 high and low group.
Differentially expressed proteins in TCGA cohort was detected according to status of CSMD1 expression. 0.2 was used as a cutoff value for log ratio. q<0.05 was considered as statistically significant

Gene	Cytoband	μ in (A) CSMD1 high	μ in (B) CSMD1 low	σ in (A) CSMD1 high	σ in (B) CSMD1 low	Log Ratio	q-Value	Higher expression in
**KIT**	4q12	0.37	-0.11	0.65	0.59	0.48	3.29e-12	A) CSMD1 high
**BRAF**	7q34	0.34	-0.13	0.76	0.67	0.47	1.59e-9	A) CSMD1 high
**RPS6KB1_PT389**	6q21	0.35	-0.06	0.93	0.81	0.41	1.385e-5	A) CSMD1 high
**ACACA**	17q12	0.18	-0.16	0.45	0.50	0.35	1.01e-11	A) CSMD1 high
**TSC2**	16p13.3	0.12	-0.16	0.35	0.41	0.28	8.36e-12	A) CSMD1 high
**PTEN**	10q23.31	0.15	-0.11	0.34	0.36	0.26	3.29e-12	A) CSMD1 high
**MAP2K1**	15q22.31	0.25	-0.01	0.57	0.47	0.26	3.627e-6	A) CSMD1 high
**GSK3A**	19q13.2	0.16	-0.08	0.43	0.32	0.24	4.92e-9	A) CSMD1 high
**GSK3B**	3q13.33	0.16	-0.08	0.43	0.32	0.24	4.92e-9	A) CSMD1 high
**FASN**	17q25.3	0.15	-0.08	0.31	0.33	0.23	2.01e-11	A) CSMD1 high
**RB1_PS807_S811**	5q21-q22	0.17	-0.05	0.63	0.65	0.22	1.543e-3	A) CSMD1 high
**PRKCA**	17q24.2	0.09	-0.13	0.49	0.57	0.22	1.134e-4	A) CSMD1 high
**PRKCA_PS657**	4q24	0.14	-0.07	0.53	0.58	0.21	4.750e-4	A) CSMD1 high
**TP53BP1**	15q15.3	0.13	-0.08	0.48	0.48	0.21	7.135e-5	A) CSMD1 high
**EIF4EBP1_PT37_T46**	5q21-q22	0.19	-0.02	0.71	0.60	0.21	4.450e-3	A) CSMD1 high
**SYK**	9q22.2	-0.22	0.22	0.45	0.53	-0.44	1.29e-16	(B) CSMD1 low
**CAV1**	7q31.2	-0.12	0.30	0.48	0.82	-0.42	1.59e-9	(B) CSMD1 low
**MAPK1_PT202_Y204**	5q21-q22	-0.17	0.18	0.94	0.92	-0.34	5.445e-4	(B) CSMD1 low
**MAPK3_PT202_Y204**	5q21-q22	-0.17	0.18	0.94	0.92	-0.34	5.445e-4	(B) CSMD1 low
**STAT3_PY705**	19q13.33	-0.11	0.12	0.46	0.48	-0.23	6.509e-6	(B) CSMD1 low
**RPS6_PS235_S236**	5q21-q22	-0.11	0.12	0.38	0.47	-0.23	3.78e-7	(B) CSMD1 low
**EGFR_PY1068**	7q31	0.07	0.29	0.51	1.02	-0.21	0.0105	(B) CSMD1 low

Supplementary Table 4 Upstream regulators predictively activated or inhibited in CSMD1 high group in comparison to CSMD1 low group in TCGA cohort. Upstream regulators whose z-score>2 or <-2 according to IPA was shown.

Upstream Regulator	Expr Log Ratio	Molecule Type	Predicted Activation State	Activation z-score	p-value	Target Molecules in Dataset
ETV6-RUNX1		fusion gene/product	Activated	3,679	0,000243	ALOX5,ALOX5AP,CALN1,CCL5,CD48,CHI3L2,CLIC1,CYBA,CYBB,ELF4
EOMES	-0,53	transcription regulator	Activated	3	0,00115	ANXA1,CCKBR,DHRS2,HOXA10,HOXA13,HOXA9,PDPN,PITX1,ZCCHC12
TFRC	-0,09	transporter	Activated	2,646	0,00349	ANKRD22,ANXA2,CD300A,ITGB2,LRRC25,MS4A6A,RGS1
COL18A1	-0,4	other	Activated	2,621	0,0604	CCL2,CHGA,ICAM1,ID3,ITGB2,PLAU,SERPINE1
IL1RN	-0,87	cytokine	Activated	2,563	0,00681	CD44,CIITA,CTSS,GBP1,HLA-DQB1,ICAM1,LGALS9,SERPINE1,SLC15A3,SP100
RARA	0,08	ligand-dependent nuclear receptor	Activated	2,449	0,256	ALOX5,CD14,CD44,CD7,CP,CRH,CTSS,GDF15,HOXA5,RASGRP4
SAFB	-0,03	other	Activated	2,333	0,000302	CCL5,CD74,CNTNAP2,CXCL10,HLA-DPA1,HLA-DPB1,HLA-DRA,HMOX1,HOXC6,L1CAM
estrogen receptor		group	Activated	2,242	3,62E-06	ANXA1,C3,CALB2,CAPG,CAV1,CD44,CD68,CD99,CDH12,CDH13
EFNA5	0,68	kinase	Activated	2,236	0,0649	APOBEC3G,FOSL1,FOXD3,ITGB4,KLK7
EFNA4	-0,95	kinase	Activated	2,236	0,0705	APOBEC3G,FOSL1,FOXD3,ITGB4,KLK7
EFNA3	0,26	kinase	Activated	2,236	0,0705	APOBEC3G,FOSL1,FOXD3,ITGB4,KLK7
PARP1	-0,01	enzyme	Activated	2,2	0,0404	GDF15,IL10,NELL2,PVALB,RAPGEF4
IL2	-0,03	cytokine	Inhibited	-2,049	0,00356	CACNA1E,CACNG3,CARD9,CASP1,CCL2,CD44,CD69,CD86,CDK5R1,CISH
NFkB (complex)		complex	Inhibited	-2,088	0,00053	ADGRE1,AMPH,BCL2A1,C3,CCL2,CCL5,CD44,CD86,CIITA,CRHR1
IL18	-1,17	cytokine	Inhibited	-2,126	0,0214	CCL2,CHRNA7,CXCL10,HAVCR2,ICAM1,IL10,TIMP1,VCAM1
CEBPA	-0,95	transcription regulator	Inhibited	-2,144	0,00126	ALOX5AP,ANXA1,BCL2A1,C3,CD7,CHI3L1,EEF1A2,GBP1,GPR84,HAMP
SPP1	-1,11	cytokine	Inhibited	-2,148	0,274	CCL2,CD163,CD44,PLAU,VIM
FOS	-0,79	transcription regulator	Inhibited	-2,156	0,044	CASP4,CCL2,CCL5,CD44,DIO2,HAMP,ICAM1,SERPINE1,VCAM1

MAP2K1/2		group	Inhibited	-2,163	0,15	C3,CHI3L1,FOSL1,IL10,VIM
TWIST1	-0,38	transcription regulator	Inhibited	-2,177	0,0373	C3,CD44,COL1A1,LEFTY2,S100A4,VIM
IL1B	-0,52	cytokine	Inhibited	-2,177	0,00176	ACAN,APOC2,BCL2A1,C3,CCL2,CCL5,CHI3L1,CIITA,COL1A1,CTSS
P38 MAPK		group	Inhibited	-2,181	0,00395	ANK1,CCL2,CCL5,CD44,CD86,CRHR1,CXCL10,DIO2,GBP1,HMOX1
Ifn gamma		complex	Inhibited	-2,183	0,00334	CXCL10,CYBB,GBP1,HLA-DRA,ICAM1
SREBF1	-0,12	transcription regulator	Inhibited	-2,185	0,00755	APOBEC3F,C5AR1,CD14,CFI,FBXO32,GPNMB,HMOX1,IFI30,MYOD1,PLEKHA4
CEBPB	-0,81	transcription regulator	Inhibited	-2,2	0,0468	C3,CCL5,CCR5,GNLY,HAMP,IL10,LY96,STAR,TLR8
RELA	-0,22	transcription regulator	Inhibited	-2,205	0,00854	ALOX5AP,CAV1,CCL2,CCL5,CD44,CIITA,COL1A1,CXCL10,ICAM1,IL10
ETS1	0,33	transcription regulator	Inhibited	-2,213	0,0207	CASP1,CD44,CD69,FOXD1,NCF1,PLAU,RUNX1,SP100,TBXAS1
PTGS2	-0,08	enzyme	Inhibited	-2,219	0,0023	ANXA1,ANXA2,CCL2,CCL5,CXCL10,ICAM1,ITGAL,ITGB4,MSR1
TIFA	-0,53	other	Inhibited	-2,219	0,000563	CCL2,CCL5,CXCL10,HAMP,ICAM1
JUNB	-0,72	transcription regulator	Inhibited	-2,219	0,00911	CASP4,CD44,FOSL1,HMOX1,ITGB4,PLAUR,SERPINE1
EBI3	-0,97	cytokine	Inhibited	-2,306	3,57E-08	CD86,CIITA,HLA-DMA,HLA-DMB,HLA-DPA1,HLA-DQA1,HLA-DRA,ICAM1,IL10,NFATC1
CSF2	-0,03	cytokine	Inhibited	-2,354	0,00652	CCL2,CD14,CD163,CD33,CD69,CIITA,CTSC,CYBB,FCGR2B,ICAM1
ERK		group	Inhibited	-2,371	0,000433	APOBEC3G,CCL2,CDK5R1,COL1A1,CXCL10,DIO2,FOSL1,GDF15,HOXA1,ICAM1
EGFR	0,39	kinase	Inhibited	-2,401	0,238	CAV1,CCL2,CHI3L1,GBP1,ICAM1,PLAU,PLAUR,VIM
SMARCA4	0,08	transcription regulator	Inhibited	-2,424	2,26E-08	APOBEC3G,AZGP1,BATF,CALB2,CARD16,CASP1,CCL2,CD44,CD74,CIITA
KDM3A	0,07	transcription regulator	Inhibited	-2,433	0,00252	CCL2,CCN1,FLNC,GDF15,HMOX1,SERPINE1
CCL5	-1,12	cytokine	Inhibited	-2,433	0,0204	C5AR1,CCL2,CD163,CD44,EMP1,PLAUR
NEDD9	-0,13	other	Inhibited	-2,433	0,0143	ALOX5,GDF15,MMP14,PLAC8,SERPINE1,VIM
IL5	-0,05	cytokine	Inhibited	-2,449	0,0444	CCL2,CD69,CIITA,CTSC,FCGR2B,SLC1A5
MAPK14	0,03	kinase	Inhibited	-2,449	0,00522	ALOX5AP,CCL2,ICAM1,IL10,SPP1,VCAM1
NFKB1	-0,33	transcription regulator	Inhibited	-2,449	0,000347	CASP4,CCL2,CCL5,CCN1,CD44,CHI3L1,CIITA,COL1A1,CXCL10,GFRA1

CD36	-0,28	transmembrane receptor	Inhibited	-2,449	0,0227	CCL5,ICAM1,MMP14,PLAU,PLAUR,SERPINE1
ERK1/2		group	Inhibited	-2,543	0,2	C3,CCL2,CCL5,CD44,CD86,CRHR1,ICAM1,MAP4K1,TIMP1,VCAM1
PRKCD	-0,46	kinase	Inhibited	-2,569	0,0188	BCL2A1,CIITA,COL1A1,CXCL10,FOSL1,GEM,GPRC5A,ICAM1,IL2RG,OSR2
IL6	-0,76	cytokine	Inhibited	-2,57	0,0022	ABCC3,C3,CCL2,CD163,CEBPD,CXCL10,HAMP,LY96,LYZ,MMP7
CD14	-1,21	transmembrane receptor	Inhibited	-2,592	2,24E-05	CCL2,CCL5,CXCL10,ICAM1,IL10,TLR2,VCAM1
CD40LG	-0,48	cytokine	Inhibited	-2,608	0,00447	CCL2,CD69,CD86,CXCL10,EGR4,FCGR2B,HLA-DQA1,HLA-DQA2,ICAM1,IL10
Akt		group	Inhibited	-2,616	0,217	BCL2A1,CD44,FOSL1,MMP14,MUC1,SERPINE1,VCAM1
CD40	-0,74	transmembrane receptor	Inhibited	-2,638	0,012	APOBEC3G,BCL2A1,CCL2,CCL5,CD44,CD86,FCGR2B,ICAM1,IL10,TLR2
IL27	-0,54	cytokine	Inhibited	-2,74	1,46E-08	ADGRE1,CCL2,CCL5,CD14,CD163,CD69,CD86,CIITA,CXCL10,HLA-DMA
STAT3	-0,27	transcription regulator	Inhibited	-2,745	0,00854	C5AR1,CASP4,CCL2,CD44,CD86,CHI3L1,COL1A1,CXCL10,DLEC1,HAMP
IFNL1	-0,01	cytokine	Inhibited	-2,786	0,0343	BST2,CXCL10,GBP1,GBP5,HERC5,IL10,SLC15A3,SP100
IL1A	-0,66	cytokine	Inhibited	-2,824	0,0026	CCL2,CCL5,CD44,CXCL10,FOSL1,ICAM1,PDCD1LG2,PLAU,SERPINA1,SERPINA3
TLR4	-0,2	transmembrane receptor	Inhibited	-2,838	0,00127	C3,CCL2,CCL5,CCR5,CD44,CD86,CXCL10,ICAM1,IL10,IL18
Jnk		group	Inhibited	-2,961	0,044	CCL2,CCL5,CD86,GDF15,ICAM1,MMP14,SERPINE1,TIMP1,VCAM1
JUN	-0,66	transcription regulator	Inhibited	-3,077	0,0548	CASP4,CCL2,CD44,CXCL10,DIO2,FOSL1,HMOX1,ICAM1,IL10,ITGB4
REST	-0,28	transcription regulator	Inhibited	-3,102	5,42E-08	CARTPT,GJD2,GLRA1,GRM1,INA,KCNQ3,SCG2,SNAP25,SYN1,SYP
SYK	-0,94	kinase	Inhibited	-3,104	0,000222	CCL2,CCL5,CD44,CD69,CDH12,CXCL10,FCER1G,FOSL1,GPLD1,ICAM1
IL17A	0	cytokine	Inhibited	-3,117	0,00377	ACAN,BCL2A1,CCL2,CD14,CD163,CD68,COL1A1,ICAM1,TIMP1,VCAM1
IFNA2	0,07	cytokine	Inhibited	-3,121	0,0157	BST2,CISH,CXCL10,GBP1,GBP2,HERC5,IL10,LILRB2,LILRB4,SP100
TGFB1	-0,87	growth factor	Inhibited	-3,125	0,0743	ACAN,ALOX5,CCL2,CCL5,CD163,CD44,CD86,CHI3L1,COL1A1,FAP
TGM2	-0,23	enzyme	Inhibited	-3,579	0,0066	AFF2,BCL2A1,C3,C5AR1,CCL2,CD300C,CD74,CD86,CXCL10,FCER1G
TNF	-0,75	cytokine	Inhibited	-3,651	0,00175	ABCC3,APOC2,BCL2A1,BST2,C3,CCK,CCL2,CCL5,CD163,CD44
STAT1	-0,24	transcription regulator	Inhibited	-4,319	4,33E-07	APOBEC3G,APOL4,BST2,C1R,C4A/C4B,CCL2,CD14,CIITA,CTSS,CXCL10
IFNG	-0,16	cytokine	Inhibited	-5,56	1E-08	AIF1,BCL2A1,BST2,C1QA,C1QB,C1QC,C3,CAMK4,CASP1,CCL2

Supplementary Table 5 Notable molecules are presented with expression log ratio, location type(s), their role as biomarkers as well as drugs that target them. Expression log ratios>1 and <-1 were used as thresholds.

Gene Symbol	Log Ratio	Location	Type(s)	Biomarker Application(s)	Drug(s)
CACNG2	2,74	Plasma Membrane	ion channel	diagnosis	
SULT4A1	1,79	Cytoplasm	enzyme	efficacy	
DCC	1,7	Plasma Membrane	transmembrane receptor	prognosis	
ABCC8	1,59	Plasma Membrane	transporter	efficacy	acetohexamide,chlorpropamide,gliclazide,glimepiride,glipizide,gliquidone,glyburide,minoxidil,mitiglinide,nateglinide,repaglinide,tolazamide,tolbutamide
KLK7	1,54	Extracellular Space	peptidase	diagnosis	
CRH	1,53	Extracellular Space	cytokine	efficacy	
MYOD1	1,53	Nucleus	transcription regulator	prognosis	
HTR2C	1,51	Plasma Membrane	G-protein coupled receptor	safety	agomelatine,apomorphine,aripiprazole,asenapine,blonanserin,buspirone,caffeine/ergotamine,chlorprothixene,clomipramine,cyproheptadine,dexamethasone/olanzapine,dihydroergotamine,doxepin,epinastine,ergotamine,fenfluramine,fenfluramine/phentermine,fluoxetine/olanzapine,lisuride,lorcaserin,methysergide,mirtazapine,nefazodone,olanzapine,opipramol,paliperidone,pimavanserin,quetiapine,risperidone,sertindole,trazodone,vabicaserin,ziprasidone
OPCML	1,42	Plasma Membrane	other	diagnosis	
VIP	1,41	Extracellular Space	other	efficacy	

CHRNA7	1,4	Plasma Membrane	transmembrane receptor	prognosis	ABT-089,acetylcholine,amobarbital,arecoline,atracurium,cisatracurium,D-tubocurarine,doxacurium,enflurane,isoflurane,JNJ-39393406,lobeline,mecamylamine,metocurine,mivacurium,nicotine,pancuronium,pipecuronium,rapacuronium,rocuronium,succinylcholine,varenicline,vecuronium
CACNA1G	1,35	Plasma Membrane	ion channel	diagnosis	cinnarizine,ethosuximide,flunarizine,mibefradil,trimethadione,zonisamide
TAC1	1,35	Extracellular Space	other	diagnosis	rolapitant
NRG1	1,33	Plasma Membrane	growth factor	diagnosis,response to therapy	
KCNJ11	1,28	Plasma Membrane	ion channel	efficacy	acetohexamide,chlorpropamide,glimepiride,glimepiride/pioglitazone,glimepiride/rosiglitazone,glipizide,glyburide,minoxidil,nateglinide,phentolamine,repaglinide,thiamylal,tolazamide,tolbutamide
SFRP2	1,25	Plasma Membrane	transmembrane receptor	diagnosis	
CDH13	1,18	Plasma Membrane	other	diagnosis,disease progression,prognosis	
CACNA2D3	1,18	Plasma Membrane	ion channel	prognosis	pregabalin
CACNA2D2	1,15	Plasma Membrane	ion channel	disease progression	alpha 2 delta calcium channel agonist,amiodarone,bepridil,gabapentin,isradipine,pregabalin
HS3ST2	1,14	Cytoplasm	enzyme	diagnosis	
LINGO2	1,13	Extracellular Space	other	safety	
SLIT2	1,12	Extracellular Space	other	diagnosis	
GRM1	1,12	Plasma Membrane	G-protein coupled receptor	efficacy	fasoracetam
F5	1,11	Extracellular Space	other	diagnosis	antithrombin alfa,drotrecogin alfa
KLK5	1,09	Extracellular Space	peptidase	diagnosis,prognosis	
PCDH11Y	1,06	Other	other	diagnosis	

FOXG1	1,03	Nucleus	transcription regulator	prognosis	
HOXA9	-1,01	Nucleus	transcription regulator	diagnosis	
ALOX5	-1,03	Cytoplasm	enzyme	diagnosis,efficacy	balsalazide,benoxaprofen,diclofenac,diclofenac/misoprostol,diclofenac/omeprazole,diethylcarbamazine,ebselen,masoprocol,meclofenamic acid,mesalamine,nordihydroguaiaretic acid,sulfasalazine,TA 270,zileuton
RNASE3	-1,03	Extracellular Space	enzyme	efficacy	
APOC2	-1,03	Extracellular Space	transporter	efficacy	
SFRP4	-1,04	Plasma Membrane	transmembrane receptor	diagnosis	
DIRAS3	-1,04	Plasma Membrane	enzyme	diagnosis	
CD86	-1,04	Plasma Membrane	transmembrane receptor	efficacy,prognosis	abatacept,abatacept/methotrexate,belatacept
HOXC6	-1,04	Nucleus	transcription regulator	diagnosis	
CARD9	-1,06	Cytoplasm	other	diagnosis	
MIA	-1,07	Extracellular Space	other	disease progression,prognosis	
COL1A1	-1,07	Extracellular Space	other	diagnosis	collagenase clostridium histolyticum
CD68	-1,1	Plasma Membrane	other	diagnosis,efficacy	
NKX2-5	-1,11	Nucleus	transcription regulator	diagnosis	
HOXA5	-1,14	Nucleus	transcription regulator	diagnosis	
C3	-1,15	Extracellular Space	peptidase	efficacy	IgG,pegcetacoplan
PYCARD	-1,15	Cytoplasm	transcription regulator	diagnosis	
CCR5	-1,15	Plasma Membrane	G-protein coupled receptor	diagnosis,efficacy	ancriviroc,BMS-813160,maraviroc,PRO 140,vicriviroc
CD69	-1,17	Plasma Membrane	transmembrane receptor	efficacy	
FOXD3	-1,17	Nucleus	transcription regulator	diagnosis	
SERPINE1	-1,17	Extracellular Space	other	diagnosis,disease progression,efficacy,prognosis,safety	drotrecogin alfa
GDF15	-1,17	Extracellular Space	growth factor	diagnosis	

SIGLEC1	-1,18	Plasma Membrane	other	diagnosis	
MMP7	-1,18	Extracellular Space	peptidase	diagnosis	marimastat
CAV1	-1,18	Plasma Membrane	transmembrane receptor	diagnosis	
TREM1	-1,24	Plasma Membrane	transmembrane receptor	efficacy	anti-trem1
PLAU	-1,25	Extracellular Space	peptidase	disease progression,efficacy,prognosis	
FLNC	-1,27	Cytoplasm	other	diagnosis	
HAMP	-1,3	Extracellular Space	other	efficacy	lexaptepid pegol
PDCD1LG2	-1,31	Plasma Membrane	enzyme	efficacy	anti PD-L2 antibody,CA-170
NCF1	-1,34	Cytoplasm	enzyme	efficacy	
ANG	-1,35	Extracellular Space	enzyme	response to therapy	
HOXA4	-1,36	Nucleus	transcription regulator	diagnosis	
HOXB2	-1,39	Nucleus	transcription regulator	diagnosis	
RNASE2	-1,4	Cytoplasm	enzyme	diagnosis	
S100A4	-1,41	Cytoplasm	other	disease progression	
EMP3	-1,44	Plasma Membrane	other	diagnosis	
ITGB4	-1,44	Plasma Membrane	transmembrane receptor	diagnosis	
TYMP	-1,51	Extracellular Space	growth factor	diagnosis,efficacy,prognosis,response to therapy	tipiracil
CP	-1,61	Extracellular Space	enzyme	efficacy	
CHI3L1	-1,71	Extracellular Space	enzyme	diagnosis,efficacy,prognosis	
SERPINA5	-1,72	Extracellular Space	other	diagnosis	
ABCC3	-2,13	Plasma Membrane	transporter	diagnosis,prognosis	
